# Hawthorn with “homology of medicine and food”: a review of anticancer effects and mechanisms

**DOI:** 10.3389/fphar.2024.1384189

**Published:** 2024-06-10

**Authors:** Ziying Zhou, Yi Nan, Xiangyang Li, Ping Ma, Yuhua Du, Guoqing Chen, Na Ning, Shicong Huang, Qian Gu, Weiqiang Li, Ling Yuan

**Affiliations:** ^1^ Department of Pharmacy, General Hospital of Ningxia Medical University, Yinchuan, China; ^2^ College of Pharmacy, Ningxia Medical University, Yinchuan, China; ^3^ Key Laboratory of Ningxia Minority Medicine Modernization Ministry of Education, Ningxia Medical University, Yinchuan, China; ^4^ College of Traditional Chinese Medicine, Ningxia Medical University, Yinchuan, China; ^5^ Department of Chinese Medical Gastrointestinal, The Affiliated TCM Hospital of Ningxia Medical University, Wuzhong, China

**Keywords:** hawthorn, cancer, medicine food homology, anticancer active ingredients, anticancer mechanisms, enhance efficacy and reduce toxicity, novel drug delivery systems

## Abstract

Over the past few years, there has been a gradual increase in the incidence of cancer, affecting individuals at younger ages. With its refractory nature and substantial fatality rate, cancer presents a notable peril to human existence and wellbeing. Hawthorn, a medicinal food homology plant belonging to the *Crataegus* genus in the Rosaceae family, holds great value in various applications. Due to its long history of medicinal use, notable effects, and high safety profile, hawthorn has garnered considerable attention and plays a crucial role in cancer treatment. Through the integration of modern network pharmacology technology and traditional Chinese medicine (TCM), a range of anticancer active ingredients in hawthorn have been predicted, identified, and analyzed. Studies have shown that ingredients such as vitexin, isoorientin, ursolic acid, and maslinic acid, along with hawthorn extracts, can effectively modulate cancer-related signaling pathways and manifest anticancer properties via diverse mechanisms. This review employs network pharmacology to excavate the potential anticancer properties of hawthorn. By systematically integrating literature across databases such as PubMed and CNKI, the review explores the bioactive ingredients with anticancer effects, underlying mechanisms and pathways, the synergistic effects of drug combinations, advancements in novel drug delivery systems, and ongoing clinical trials concerning hawthorn’s anticancer properties. Furthermore, the review highlights the preventive health benefits of hawthorn in cancer prevention, offering valuable insights for clinical cancer treatment and the development of TCM with anticancer properties that can be used for both medicinal and edible purposes.

## 1 Introduction

Cancer represents a grave global public health concern (Siegel et al., 2023) and has evolved into a primary cause of mortality worldwide (Sung et al., 2021). Recent data from the International Agency for Research on Cancer of the World Health Organization revealed that in 2022, there were 20 million new cancer cases globally, resulting in 9.7 million deaths. Lung cancer tops the list as the most fatal type of cancer worldwide, followed by colorectal cancer, liver cancer, breast cancer, and gastric cancer (World Health Organization, 2024). The emergence of cancer is intricately connected to both natural environmental factors and social environmental factors (Wogan et al., 2004; Danaei et al., 2005). Improper diets, imbalanced nutritional structure, obesity, and excessive exposure to air and dietary pollutants all significantly contribute to the advancement of cancer (Wright and Simone, 2016; Ksouri, 2019; Turner et al., 2020). The development of cancer is a gradual process that unfolds over an extended period. In many cases, cancers do not exhibit typical early symptoms, leading to diagnoses being made at advanced stages and ultimately resulting in a poor prognosis (Minicozzi et al., 2018). Presently, the main therapeutic strategies for cancer encompass surgery, radiotherapy, chemotherapy, immunotherapy (Gotwals et al., 2017; Igarashi and Sasada, 2020; Wahida et al., 2023), and small-molecule targeted drugs (Pérez-Herrero and Fernández-Medarde, 2015; Lee et al., 2018; Liu et al., 2021). Nevertheless, the conventional cancer therapies mentioned above may result in cancer recurrence and metastasis, as well as the potential risk of drug resistance (Ellis and Hicklin, 2009). Additionally, these therapies frequently induce toxic side effects, presenting significant challenges in terms of patient tolerance and compliance (Kroschinsky et al., 2017; Couzin-Frankel, 2022; Sedano et al., 2022; Mahmoudian et al., 2024). Therefore, it is crucial to investigate novel cancer treatment approaches and improve the predictability of cancer prevention.

TCM represents the culmination of countless years of healthcare wisdom and practical experience for the Chinese nation. In the field of cancer research, TCM has made significant contributions (Yan et al., 2017; Wang K. et al., 2021; Zhang X. et al., 2021), making it a promising area for addressing cancer challenges in the future. Medicinal and edible Chinese medicines are essential components of TCM (Chen, 2023), combining the concepts of food and medicine to create a unique treatment and diet regimen that aims to harmonize the yin and yang balance in the body. These medicines offer numerous benefits, including high safety levels, minimal side effects, flexible dosage forms, good patient compliance, and the ability to target precancerous lesions (Hou and Jiang, 2013; Jiang et al., 2023; Zhong et al., 2023). Recent studies have confirmed the anticancer properties of extracts and purified bioactive monomer components present in various medicinal and edible Chinese medicines (Huang et al., 2023). When used in combination with other chemotherapeutic drugs, these Chinese medicines have shown the ability to enhance drug efficacy, mitigate adverse reactions, overcome drug resistance, and notably enhance patients’ quality of life (Chen G. Q. et al., 2023; Lu et al., 2023). The safety and effectiveness of medicinal and edible Chinese medicines have provided a distinct advantage and valuable guidance in the realm of cancer prevention and treatment. These medicines have emerged as a new strategy for cancer treatment, significantly contributing to human health (Chen, 2023).

Hawthorn (*Crataegus pinnatifida*), known as “Shanzha” (in Chinese), is a member of the family Rosaceae and genus *Crataegus*. This plant is valued for its dual purpose as a medicinal and edible Chinese herb, with a rich history of medicinal applications that have earned it widespread acclaim for its therapeutic benefits (Li R. et al., 2023; Cui et al., 2024). In the realm of TCM, the efficacy of treatments is often attributed to the effective ingredients within them. Hawthorn stands out due to its rich phytochemicals and corresponding pharmacological effects (Ke et al., 2022). Extensive research has identified approximately 253 phytochemical ingredients in hawthorn, including flavonoids, triterpenoids, lignans, phenylpropanoids, and steroids. These ingredients have demonstrated various pharmacological effects such as antihypertensive, lipid-lowering, cardiotonic, digestion-stimulating, appetizer, antibacterial, and anticancer properties (Zhang L. L. et al., 2020; Li R. et al., 2023). Hawthorn-based drugs with proven therapeutic effects are available on the market in various dosage forms, commonly used to treat conditions related to the digestive system, cardiovascular system, cerebrovascular system, and reproductive system. For instance, YiXinTong preparation utilizes flavonoids from hawthorn leaves to exhibit pharmacological effects like inhibiting or scavenging oxygen free radicals, improving microcirculation, and protecting against inflammatory damage, effectively addressing cardiovascular diseases (Guo et al., 2023). Additionally, the HongHe Women’s Cleansing Solution, with hawthorn sperm as its main component, can regulate the body’s immune function through various targets and pathways, reducing oxidative stress, inhibiting the inflammatory response, and providing anti-infective effects (Jia et al., 2023). However, to date, no drugs utilizing hawthorn as a raw material with a definite anticancer effect have been commercially available. Therefore, integrating current anticancer research on hawthorn extraction and effective monomer ingredients, clarifying primary bioactive substances responsible for its anticancer properties, and delving into the anticancer mechanisms are of great significance for promoting the research and development of hawthorn anticancer drugs, accelerating their listing process, and further tapping the practical value of hawthorn. This move is expected to bring new ideas and methods to the field of cancer treatment and bring the gospel to the majority of patients.

**FIGURE 1:**
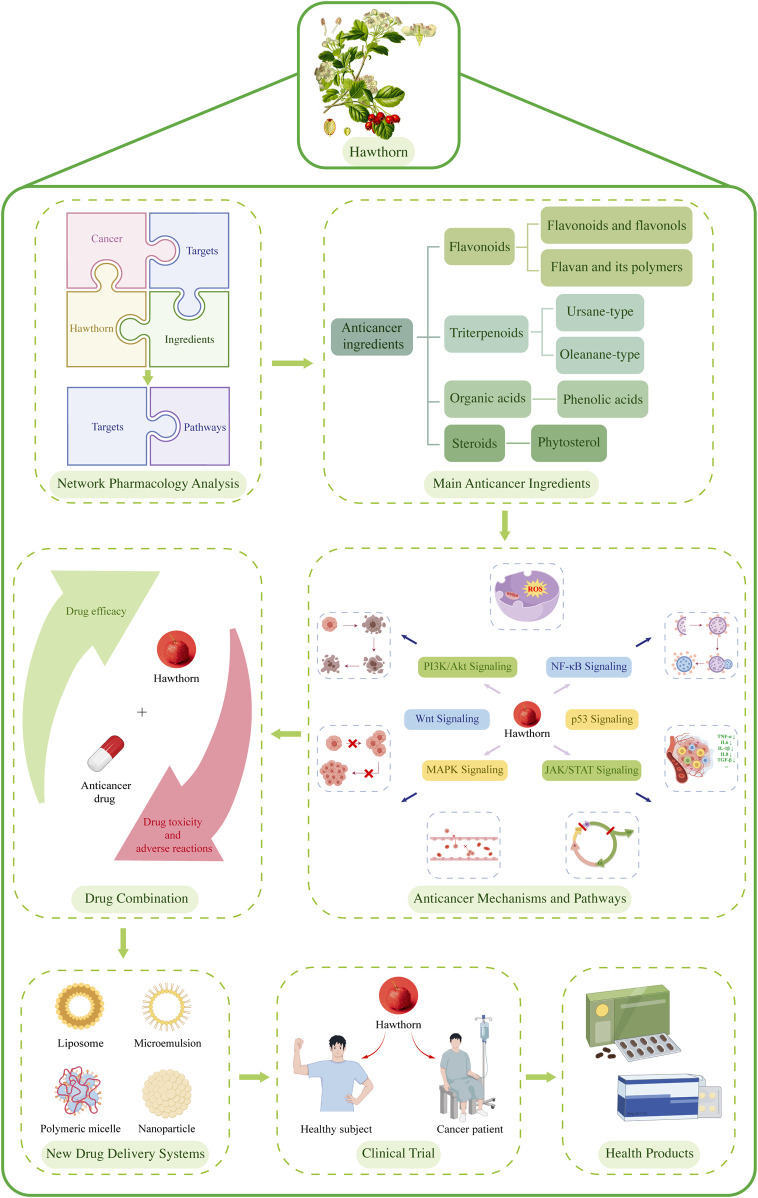
The research process of this review (some of the contents in this figure are drawn by Figdraw, http://www.figdraw.com/).

This review aims to analyze the anticancer effect of hawthorn. Initially, network pharmacology analysis was used to determine its efficacy. Subsequently, a comprehensive analysis was conducted by searching databases, including PubMed and CNKI, to systematically summarize the mechanisms and action pathways of hawthorn extracts and its main anticancer ingredients in treating various types of cancer. The review clearly demonstrates that combination therapy can enhance the sensitivity of chemotherapy drugs, reverse multidrug resistance, and significantly reduce the toxic and side effects of chemotherapy drugs on human normal tissues and organs. Furthermore, it highlights the potential of new drug delivery systems to improve bioavailability and enhance efficacy. This review also outlines the current limitations in clinical trials related to hawthorn’s anticancer properties and introduces its broad application in health food, aiming to promote the clinical development of medicinal and edible Chinese medicine hawthorn. The specific research process is depicted in Figure 1.

## 2 Network pharmacology analysis

TCM is characterized by its multi-component, multi-target, and diverse regulatory methods (Li X. et al., 2023). As network pharmacology delves deeper into the realm of TCM, the approach to drug research has shifted from focusing on single target and single component to a more holistic exploration and systematic regulation (Li and Zhang, 2013; Zhang et al., 2019; Zhu et al., 2022). By recognizing that the chemical components of TCM can affect disease-related targets through interconnected signaling pathways, network models like “disease-target-ingredient-drug” and “target-signaling pathway” have been devised (Jiashuo et al., 2022; Gan et al., 2023). These models enable a systematic and comprehensive prediction of how TCM can intervene in diseases, enriching the research landscape of TCM and providing new perspectives for modern drug research and development (Gan et al., 2023). This study utilizes network pharmacology technology to investigate the active components and targets of hawthorn, aiming to speculate on its potential indications and predict its mechanism of action.

### 2.1 Excavation of active ingredients and targets of hawthorn

The TCMSP platform (http://old.tcmsp-e.com/tcmsp.php) was utilized to search for chemical constituents in hawthorn leaves. The screening criteria of oral bioavailability ≥15% and drug-likeness ≥0.18 were applied to obtain the active ingredients and their targets. Additionally, information from literature and the SwissTargetPrediction database (http://swisstargetprediction.ch/) was incorporated to further supplement the active ingredients of hawthorn and the corresponding gene targets (Basavarajappa et al., 2023). The collected data was visually analyzed using Cytoscape 3.9.1 software, and the connectivity centrality was calculated with the assistance of the CytoNCA plug-in. To determine the key targets of hawthorn in cancer treatment, a screening condition of connectivity centrality >2 times the median value was applied. The target data was further analyzed for metabolic pathways using the David platform (https://david.ncifcrf.gov/home.jsp) (Yang J. et al., 2023). Data visualization was performed using the Chiplot platform (https://www.chiplot.online/).

### 2.2 Prediction results of network pharmacology

**FIGURE 2:**
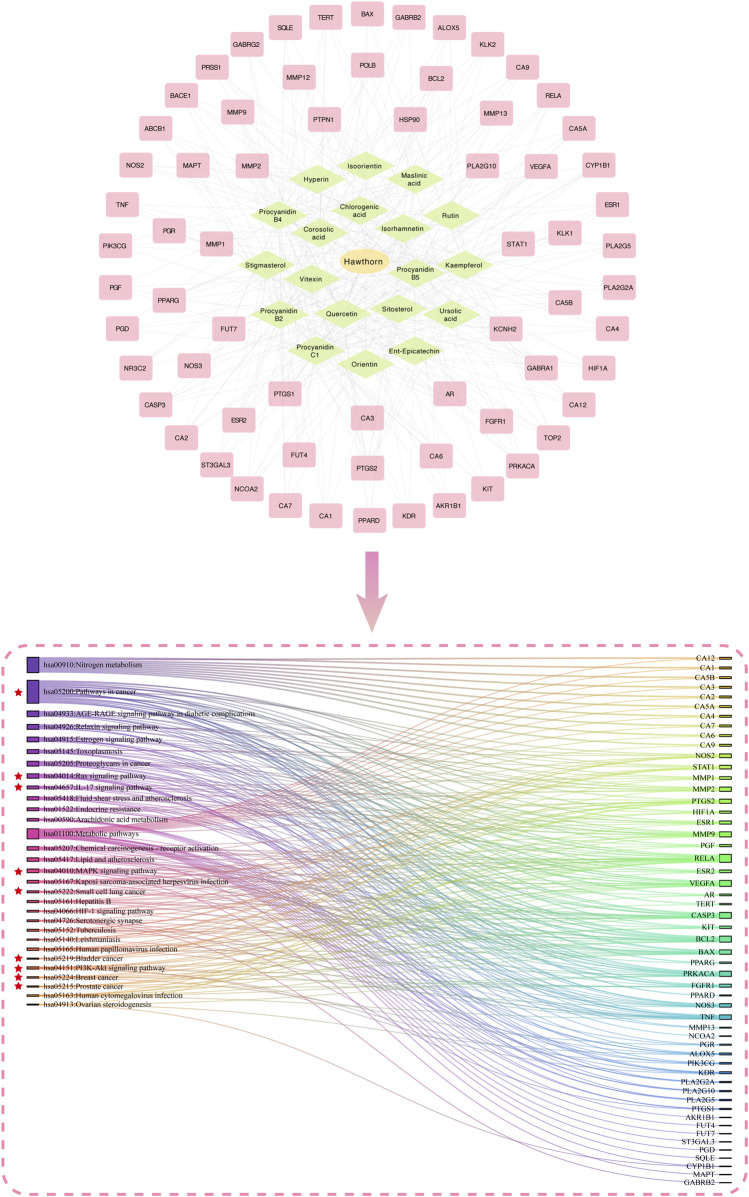
Network pharmacological analysis of related active ingredients, targets, and pathways in hawthorn. In the figure, the yellow oval represents traditional Chinese medicine, the green diamonds symbolize the active ingredients of hawthorn, and the pink rectangles indicate corresponding genes. Signal pathways enriched by core targets and closely related to tumors are identified with red asterisks.

Utilizing the TCMSP database and integrating various literature, we screened 19 active ingredients of hawthorn and pinpointed 67 core targets linked to these compounds. The pathways enriched by these core targets are closely associated with cancer. This suggests that hawthorn may possess a notable anticancer effect, with the identified active ingredients possibly being the main components of hawthorn responsible for exerting anticancer efficacy. Figure 2 visually presents the relationship network among drug, active ingredients, and targets, along with a Sankey diagram illustrating the correlation between targets and pathways.

## 3 The main anticancer bioactive ingredients in hawthorn

**FIGURE 3:**
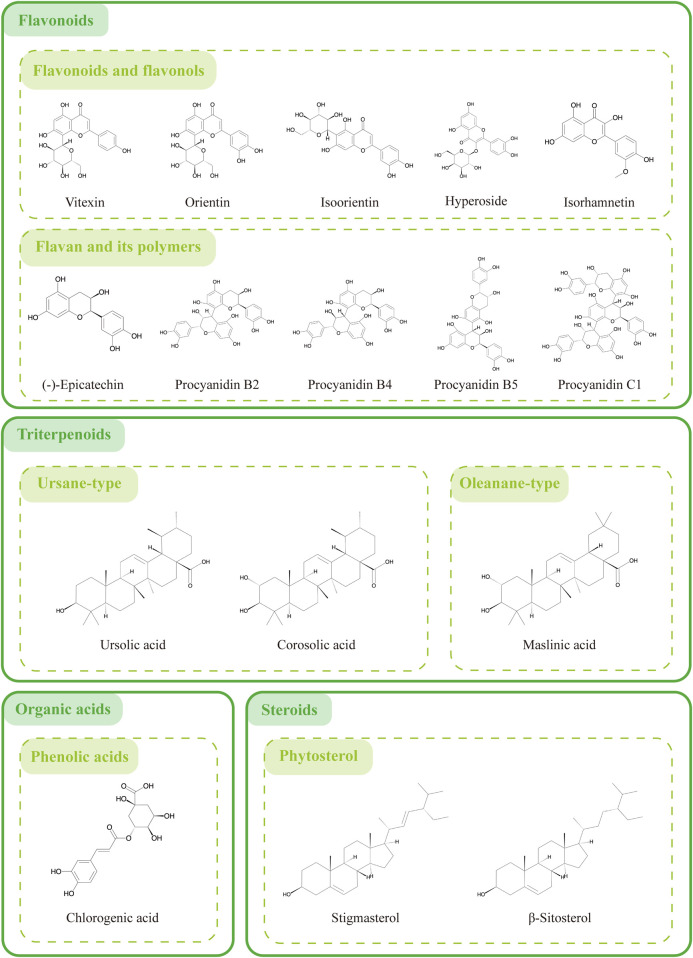
The active compounds in hawthorn.

The analysis of chemical components in plants is crucial for understanding the therapeutic potential of medicinal plants. Effective active components found in TCM serve as the material basis for disease prevention and treatment (Li et al., 2024). Hawthorn, as a plant source of bioactive compounds, has been extensively researched. Based on the current literature research findings and the prediction results of network pharmacology, we determined representative active ingredients extracted and isolated from hawthorn can play a key role in the field of anticancer and have broad development prospects. These active ingredients include flavonoids, flavan and their polymers, organic acids, as well as triterpenes and steroids (Chang et al., 2002). The chemical structures are shown in Figure 3.

### 3.1 Flavonoids

The most abundant chemical ingredient in hawthorn is a class of flavonoids and their glycosides, with apigenin and luteolin as aglycones (Rayyan et al., 2005; Zhang J. et al., 2022). Apigenin-based glycosides include vitexin and isovitexin, while luteolin-based glycosides include orientin and isoorientin. Hawthorn also contains flavonols and their glycosides, such as quercetin, kaempferol, rutin, hyperin, and isorhamnetin (Zhang et al., 2010; Jurikova et al., 2012).

In recent years, multiple studies have demonstrated that flavonoids rich in hawthorn play an anticancer role through various signal transduction pathways. For instance, vitexin has been discovered to impede the growth, blood vessel formation, and stemness of endometrial cancer cells. It achieves this by targeting the phosphoinositide-3-kinase (PI3K)/protein kinase B (Akt) signaling pathway (Liang C. et al., 2023). The mechanism of orientin involves the regulation of protein kinase C alpha (PKCα)/extracellular protein-regulated kinases (ERK)/activator protein-1 (AP-1)/signal transducer and activator of transcription 3 (STAT3) signaling pathway. Upon exposure to orientin, the activation of PKCα is suppressed, subsequently impacting the aforementioned cellular signal transduction pathways and ultimately impeding the invasion of breast cancer cells (Kim et al., 2018). Isoorientin induced programmed cell death by activating the reactive oxygen species (ROS)-mediated mitogen-activated protein kinase (MAPK)/STAT3/nuclear factor kappa B (NF-κB) pathway. Moreover, it regulated cellular migration by modulating the Akt/glycogen synthase kinase 3β (GSK-3β)/β-catenin pathway (Zhang T. et al., 2022). Hyperoside has demonstrated its efficacy in inhibiting the hypoxia-induced proliferation of cancer cells by enhancing ferrous accumulation in the adenosine monophosphate-activated protein kinase (AMPK)/heme oxygenase-1 (HO-1) axis (Chen et al., 2020). Isorhamnetin primarily acts through the peroxisome proliferator-activated receptor γ (PPARγ)/phosphatase and tensin homolog (PTEN)/Akt pathway. It suppresses cell proliferation and the transition from the G0/G1 phase to the S phase in bladder cancer by suppressing the expression of carbonic anhydrase IX (CA9), thereby diminishing tumor formation (Zhang P. et al., 2023).

### 3.2 Flavan and its polymers

These compounds are also widely distributed in hawthorn, and the basic units are (+)-catechin, (−)-epicatechin, and leucocyanidin. They can polymerize with each other to form dimers like procyanidin B2, B4, and B5, as well as trimers like procyanidin C1 (Jurikova et al., 2012).

Epidemiological studies have demonstrated that (−)-epicatechin and proanthocyanidins exhibit strong resistance against various types of cancer and can produce therapeutic effects through different mechanisms. Research conducted on mice has unveiled that (−)-epicatechin inhibits the proliferation, migration, and invasion of breast cancer cells, resulting in reduced tumor size and ultimately enhancing the survival rate of cancer-afflicted mice (Pérez-Durán et al., 2023). Furthermore, antiproliferative and apoptotic effects have been demonstrated by procyanidin B2, which induces autophagy by regulating the Akt/mechanistic target of the rapamycin (mTOR) signaling pathway (Li Y. et al., 2021). Procyanidin C1 exhibits anticancer properties by inducing DNA damage, arresting the cell cycle, and augmenting the expression of checkpoint kinases (Koteswari et al., 2020).

### 3.3 Triterpenoids

The pentacyclic triterpenoids represented by maslinic acid, corosolic acid, oleanolic acid, and ursolic acid are the primary triterpenoids found in hawthorn (López-Hortas et al., 2018; Zhang J. et al., 2022). Among them, ursolic acid has the highest concentration. Research conducted by (Liao et al., 2022) reveals that the progression of cancer heavily relies on the stemness and metastasis of cancer cells. Ursolic acid effectively obstructs the stemness of cancer cells by significantly reducing the expression of stemness biomarkers. Besides, it modifies the migration and invasion abilities of the cancer cells by regulating the MAPK-ERK/vascular endothelial growth factor (VEGF)/matrix metalloproteinase-9 (MMP-9) signaling pathway, and polyamine metabolism (Zong et al., 2022).

Lately, researchers have shown increased interest in the potential anticancer properties of corosolic acid. Studies have demonstrated that corosolic acid possesses the ability to diminish the level of cyclin-dependent kinase 19 (CDK19)-mediated O-GlcNAcylation within liver cancer cells, thereby impeding the advancement of cancer (Zhang C. et al., 2021). Additionally, corosolic acid has been observed to reduce invasion and chemoresistance in cancer cells by inducing oxidative stress in mitochondria and liposomes (Jin et al., 2021).

Maslinic acid frequently appears alongside its isomeric counterpart, corosolic acid. It exhibits remarkable inhibitory properties against cancer cells and achieves this by inducing apoptosis through the regulation of the AMPK/mTOR signaling pathway (Wei et al., 2019). Furthermore, experimental analyses conducted *in vitro* have shown that maslinic acid can exert toxic effects on cancer cells by upregulating the expression of DNA damage and repair-related proteins (Lu et al., 2020).

### 3.4 Organic acids

The content of organic acids in hawthorn’s active ingredients is second only to flavonoids, and they play a vital role in the prevention and treatment of cancer. Among these organic acids, chlorogenic acid stands out for its notable anticancer properties. It possesses the capability to hinder the epithelial-mesenchymal transition (EMT) of cancer cells, lessen their fluidity and invasiveness, and impede their growth (Xue W. et al., 2023). Additionally, chlorogenic acid effectively suppresses the nuclear transcription of NF-κB p65 by disrupting the NF-κB/EMT signaling pathway. This contributes to the prevention of cancer cell metastasis and fosters anticancer immunity (Zeng et al., 2021).

### 3.5 Steroids

β-sitosterol and stigmasterol are two steroidal compounds discovered in hawthorn. Recent research has shown that β-sitosterol possesses the capability to hinder the progression and infiltration of colorectal cancer cells by impeding the wingless/integrated (Wnt)/β-catenin pathway (Gu et al., 2023). Stigmasterol has been observed to activate protective autophagy in gastric cancer cells through the inhibition of the Akt/mTOR pathway (Zhao et al., 2021).

## 4 Anticancer effects and underlying mechanisms of hawthorn-derived compounds or extracts

Bioactive ingredients derived from TCM serve as the foundation for investigating the pharmacodynamic substances, exploring the pharmacological effects, and elucidating the mechanism of action. Phytochemical separation is a common research method (Borjigin et al., 2023). Through the extraction, separation and spectroscopy analysis of TCM, the chemical composition and structural information existing in TCM can be elucidated (Zhang W. J. et al., 2020). On this basis, combined with experimental models such as cells and animals, researchers can verify the efficacy of Chinese medicine components (Tang, 2006). This systematic research method is beneficial for elucidating the key components of TCM, understanding the mechanisms of action, and establishing a strong basis for the extensive integration of TCM into modern medical practices (Bi et al., 2018).

Based on the aforementioned theory, researchers are dedicated to investigating the impact of pure compounds and extracts from TCM, such as hawthorn, on various diseases (Hsiao and Liu, 2010; Hu et al., 2013; Wang et al., 2014; Sun et al., 2021; Yao et al., 2021; Yagüe et al., 2022; Zimmermann-Klemd et al., 2022; Huang et al., 2024). The active monomer components and extracts in hawthorn serve as the foundation for its pharmacological effects. A thorough examination of these components is essential for uncovering hawthorn’s anticancer mechanism, offering significant scientific research and clinical implications (Cui et al., 2024). As more studies on hawthorn’s anticancer properties emerge in the medical field, the exploration of its anticancer mechanism continues to progress. The anticancer effects of hawthorn are primarily attributed to various mechanisms, including the inhibition of cancer cell growth through suppressing cancer cell proliferation, halting the cell cycle, triggering cancer cell apoptosis, and regulating autophagy; Hawthorn also limits the migration, invasion, and adhesion of cancer cells, thereby hindering the carcinogenesis process by obstructing the degradation of the extracellular matrix (ECM), restraining angiogenesis, regulating tumor cell EMT and the tumor microenvironment (TME); Furthermore, hawthorn averts cancer progression by inducing the generation of ROS and inhibiting the synthesis of inflammatory molecules during carcinogenesis.

### 4.1 Inhibition of cancer cell proliferation

The formidable capacity for proliferation displayed by cancer cells is among the primary factors contributing to their resistance to effective elimination. This ceaseless proliferation additionally imposes a substantial burden on the body. Therefore, the inhibition of cancer cell proliferation has arisen as a pivotal strategy in the treatment of cancer. In contemporary scientific research, the utilization of separation and purification technology is commonly employed to investigate novel compound information found in distinct medicinal sections of hawthorn. To validate its efficacy in combating cancer, experiments assessing cytotoxicity are commonly conducted (Hsiao and Liu, 2010; Zimmermann-Klemd et al., 2022).

Building on this concept, neolignans with antioxidant activity have been successfully isolated from hawthorn seeds (Huang et al., 2013a; Li L. Z. et al., 2013; Huang et al., 2013b), demonstrating significant inhibition of cancer cell growth in a dose-dependent manner. Research on the medicinal potential of hawthorn leaves revealed that methanol, acetone (Mohammedsaeed and Mohamad, 2023), and ethyl acetate extracts (Mustapha et al., 2015) exhibit notable anti-proliferative effects on cancer cells. Triterpenoids (Min et al., 2000) and total flavonoids (Tang et al., 2010; Diao et al., 2019) isolated from these extracts also showed promise in inhibiting cancer cell activity. Hawthorn buds extract displayed significant cytotoxicity in four human cancer cell lines (Rodrigues et al., 2012), while the petroleum ether extract of hawthorn stems exhibited stronger inhibition of cancer cell proliferation and promotion of apoptosis compared to water and ethanol extracts (Maldonado-Cubas et al., 2020). Triterpenoids (Ahn et al., 1998; Qiao et al., 2015), neolignans (Guo et al., 2019a; Shang et al., 2020), polyphenols (Żurek et al., 2021), total flavonoids (Zhang et al., 2004), and aromatic compounds (Guo et al., 2018; Zhao et al., 2019) found in hawthorn fruit showed potent anticancer cell proliferation and antioxidant activity. Notably, polyphenol components in hot water extract of dried hawthorn fruit were found to significantly inhibit tumor formation and reduce tumor incidence (Kao et al., 2007). In addition, the polyphenolic components in hawthorn whole plant extract have demonstrated a reduction in cell viability and reactive oxygen species formation in a dose- and time-dependent manner, indicating both cytotoxic and antioxidant properties (Belščak-Cvitanović et al., 2014). Simultaneously, hawthorn extract has a certain anti-mutation effect while causing tumor cell death (Zhu, 2012) and inhibiting its proliferation (Sun et al., 2013; Mustapha et al., 2016a).

The bioactive ingredients abundant in hawthorn have been identified as the basis for its anticancer properties. Through basic experimentation, preliminary confirmation of the intervention impact and mechanism of these chemical ingredients on cancer has been achieved. Vitexin, the primary active ingredient in hawthorn leaves, possesses the capability to influence the expression of specific genes in the signaling pathway associated with “anti-proliferation,” rendering it a potential drug for breast cancer treatment and prevention through the mediation of miRNA (Najafipour et al., 2022). Hyperoside is also highly acclaimed in the realm of cancer therapy (Peng et al., 2023), as it has been demonstrated to effectively impede the growth of cervical cancer cells by targeting the V-Myc myelocytomatosis viral oncogene homolog (C-MYC) gene (Guo W. et al., 2019). Additionally, it has exhibited promise in managing non-small cell lung cancer (NSCLC) with the T790M mutation. By upregulating the expression of forkhead box protein O1 (FoxO1), it hinders cancer cell proliferation and induces apoptosis (Hu et al., 2020). Stigmasterol, renowned for its potent biological activity, has emerged as a notable area of interest in the exploration of natural active ingredients present in hawthorn. Recent investigations have unveiled that retinoic acid-related orphan receptor C (RORC) can specifically target stigmasterol, leading to the suppression of lung cancer cell proliferation. This discovery provides a promising direction for the development of potential therapeutic strategies to combat lung cancer (Dong et al., 2021).

### 4.2 Initiation of cancer cell apoptosis

Apoptosis, also known as programmed cell death, is a crucial mechanism that regulates and examines cells by employing caspase proteolytic enzymes under specific physiological or pathological circumstances. This process efficiently eliminates non-functioning, abnormal, harmful, and misplaced cells (Carneiro and El-Deiry, 2020). Apoptotic cells demonstrate noticeable changes in morphology, including shrinkage, chromatin condensation, and the formation of apoptotic bodies (Wong, 2011). There are two primary signaling pathways responsible for inducing cell apoptosis: the extrinsic/death receptor pathway and the intrinsic/mitochondrial pathway (Ghobrial et al., 2005; Wong, 2011; Carneiro and El-Deiry, 2020).

For a long time, apoptosis has been recognized as a crucial mechanism in preventing tumor development, and many cancer treatments rely on promoting effective apoptosis (Singh and Lim, 2022). In recent years, researchers have focused on discovering cancer treatment methods that target apoptosis-related molecules to improve treatment sensitivity and specificity. When the body contains elevated levels of anti-apoptotic proteins such as B cell lymphoma-2 (Bcl-2) and decreased levels of pro-apoptotic proteins like Bcl2-associated X protein (BAX), cancer cells exhibit anti-apoptotic activity, and malignancy increases. Hence, the utilization of the pro-apoptotic effects exhibited by members of the Bcl-2 protein family has emerged as a crucial approach in the treatment of cancer (Kaloni et al., 2023). In the investigation of the anticancer properties of hawthorn’s active ingredients, the researchers discovered that chlorogenic acid (Wang et al., 2019) and hyperoside (Qiu et al., 2019) can modify the Bax/Bcl-2 ratio, resulting in a significant induction of apoptosis and displaying their therapeutic potential in cancer treatment.

Moreover, in cancer cells, the protein caspase plays a pivotal role in both triggering and executing apoptosis. The hindered activity or impaired function of caspase expedites the progression of cancer. Activating caspase activity has long been acknowledged as a significant indication of cell apoptosis and has emerged as a noteworthy strategy in the clinical treatment of cancer. Recent research has revealed that hawthorn extract and its active ingredients possess the potential to activate caspase and stimulate apoptosis. Specifically, hawthorn peel polyphenol extract and pulp polyphenol extract have been shown to induce apoptosis in breast cancer cells, operating through the mitochondrial pathway. This is evident from the increased expression of caspase-3 and caspase-9 (Li T. et al., 2013). Additionally, Hawthorn oligomeric procyanidin extracts have demonstrated the ability to enhance apoptosis and exhibit an anticancer effect by modulating the expression levels of caspase-9 in the mitochondrial pathway, caspase-8 in the death receptor pathway, and caspase-3, serving as a common downstream regulator of both pathways. This discovery offers a fresh approach for treating colon cancer patients in clinical practice (Sun et al., 2022). Hawthorn leaf extract showed the ability to enhance the apoptosis of cancer cells (Omairi et al., 2020). This effect primarily involves the exogenous apoptosis pathway and triggers caspase-8 cleavage (Mustapha et al., 2016b). Furthermore, in experiments where the ethanol extract of hawthorn was applied to liver cancer cell lines, it was observed that as the concentration of the extract increased and the duration of exposure lengthened, there was a notable suppression of cell proliferation and an increase in apoptosis. These outcomes were linked to the activation of the caspase pathway and a significant rise in the levels of intracellular cleaved-caspase3 and Bax/Bcl-2 proteins (Peng et al., 2016).

The role of the endoplasmic reticulum pathway in apoptosis is also crucial (Wong, 2011). In normal physiological conditions, amino acids are dehydrated and condensed to form peptide chains, which then enter the endoplasmic reticulum for processing and protein formation. Correctly folded proteins are secreted out of the cell by the Golgi apparatus, and protein synthesis and decomposition maintain a dynamic balance. However, specific circumstances such as elevated cellular Ca^2+^ levels, changes in redox status, decreased ATP levels, increased misfolded proteins, and excessive protein buildup can disrupt this equilibrium, leading to endoplasmic reticulum stress (Jin and Sun, 2020). Excessive stress response triggers intracellular apoptosis signals, promoting cell apoptosis. In a research study carried out by Ye et al. (2022), it was observed that isorhamnetin, an anticancer active ingredient found in hawthorn, can induce endoplasmic reticulum stress-related reactions. This is achieved by activating both the endogenous mitochondrial apoptosis pathway and the exogenous death receptor pathway, consequently provoking apoptosis in breast cancer cells. Tang et al. (2023) combined transcriptomic data with experimental results and discovered that corosolic acid can instigate endoplasmic reticulum stress by activating the mitochondrial pathway of apoptosis, leading to significant apoptosis in cell lines. These discoveries offer valuable insights into potential therapeutic approaches for cancer treatment.

### 4.3 Induction of cell cycle arrest

The regulation of cell growth, development, and differentiation is critically dependent on the cell cycle, which is highly organized and strictly controlled by a unique system to ensure the precise replication of genetic materials and cell division (Otto and Sicinski, 2017). The normal cell cycle comprises interphase, which includes the G1, S, and G2 phases, followed by the mitosis phase (M phase). The transition from one stage to the next is known as the cell cycle checkpoint (Matthews et al., 2022). In cancer cells, various cumulative mutations lead to abnormal mitotic signals, further leading to unplanned proliferation and increased chromosome number uncertainty (Malumbres and Barbacid, 2009). Cell cycle arrest enables the detection and repair of cell damage, reduces the occurrence of mutations, and ensures genome stability, thereby preventing the onset and progression of cancer. Therefore, the regulation of the cell cycle is of great significance in cancer treatment and prevention.

In recent years, as numerous scholars have made significant progress in investigating the anticancer mechanisms of hawthorn, the participation of hawthorn extract and its primary active ingredients in cell cycle regulation has been identified. The two key proteins involved in this process are cyclins and cyclin-dependent kinases (CDKs) (Matthews et al., 2022). Wen et al. (2017) specifically extracted triterpenoid-rich fraction S9 and ursolic acid from hawthorn fruit. They observed a decrease in the levels of Cyclin-D1 and CDK4 proteins when these ingredients were used to treat cells. Furthermore, the expression of p21^WAF1/CIP1^, a crucial member of the CDK inhibitor family, increased, resulting in significant cell G1 phase arrest. The homogeneous polysaccharide extracted from hawthorn can induce cell cycle arrest in S and G2/M phases by down-regulating Cyclin A1/D1/E1 and CDK-1/2 expression (Ma et al., 2020). Orientin, a compound commonly found in medicinal plant parts such as hawthorn, has shown promising anticancer properties. Research has demonstrated its ability to regulate cyclins and CDKs, effectively halting the cell cycle progression from the G0/G1 phase to the S phase (Thangaraj et al., 2019).

### 4.4 Regulation of autophagy

Autophagy is a catabolic process that is highly conserved in eukaryotes and plays a crucial role in maintaining cellular energy supply, material circulation, and the self-renewal of cells (Zhou et al., 2022). Its role in cancer development is complex and can be compared to a metaphorical “double-edged sword” (Debnath et al., 2023). In the early stages of cancer, autophagy activation contributes to normal cellular physiological metabolism and helps maintain the stability of the intracellular environment by limiting genomic damage and mutation, as well as selectively removing misfolded proteins. However, as cancer progresses to an advanced stage, autophagy is activated in the absence of nutrition and hypoxia. In this context, autophagy acts as a dynamic degradation and recycling system, breaking down macromolecules and providing nutrients for cancer cell growth (Li X. et al., 2020). Therefore, the appropriate activation and inhibition of autophagy is a potential direction for cancer treatment.

Flow cytometry analysis demonstrated that phenylpropanoid derivatives derived from hawthorn fruits activated protective autophagy in HepG2 cells and demonstrated anticancer activity (Guo et al., 2019b). In breast cancer, vitexin was found to notably enhance the expression of genes associated with autophagy, including autophagy-related 5 homolog (ATG5), Beclin-1, and microtubule-associated protein 1 light chain 3 II (LC3-II), thereby promoting autophagy and resulting in therapeutic benefits (Ghazy and Taghi, 2022).

### 4.5 Inhibition of cancer cell migration and invasion

Highly invasive cancer is characterized by the strong ability of cancer tissues to migrate to normal tissues. Cancer cells originate from the primary lesion and can invade the host’s blood and lymphatic vessels, spreading to distant parts of the body through blood vessels or body cavities. They can evade the host’s immune surveillance and reproduce, ultimately leading to new angiogenesis and metastasis (Xiong et al., 2022). During this process, the pathogenic body alters the adhesion and migration ability of cancer cells through factors such as ECM degradation, vascular factor production, EMT modulation, and TME regulation. Therefore, it is possible to inhibit cancer cell metastasis and delay the progression of cancer by targeting these mechanisms.

#### 4.5.1 Inhibition of ECM degradation

Malignant tumor cells can penetrate the ECM, grow around the basement membrane defect and ECM gap, and ultimately invade normal tissues and metastasize (Mierke, 2019). Matrix metalloproteinases (MMPs) are the key proteases in the ECM that facilitate cancer cell metastasis. Generally, increased levels of MMPs in cancer cells indicate increased malignancy (Almutairi et al., 2023). Recent research on the active ingredients found in hawthorn has demonstrated that isoorientin (Huang et al., 2020) and isorhamnetin (Wang et al., 2022) have the ability to inhibit matrix metallopeptidase 2 (MMP-2) and MMP-9, thereby obstructing cellular migration. These discoveries imply that the previously mentioned ingredients exhibit potential as prospective anticancer medications.

#### 4.5.2 Inhibition of tumor angiogenesis

The growth of malignant tumors is closely linked to the oxygen and blood supply provided by neovascularization (Kerbel, 2008). This biological process is mediated by a protein called VEGF (Guyot et al., 2017). Vascular endothelial growth factor A (VEGFA) belongs to the VEGF family and can enhance vascular permeability, accelerate ECM degradation, facilitate the migration and multiplication of vascular endothelial cells, and induce the formation of new blood vessels. Recent scientific investigations have provided evidence indicating that vitexin can diminish the expression of VEGFA and VEGFR2 and reduce the carcinogenic effects of ovarian cancer (Zhao et al., 2020).

#### 4.5.3 Regulation of EMT in cancer cells

During cancer progression, the process of EMT causes polarized epithelial cells to acquire mesenchymal characteristics (Ribatti et al., 2020). This transition is marked by a downregulation in the levels of E-cadherin expression, coupled with an upregulation in the levels of vimentin and N-cadherin expression (Rezaei et al., 2012). Consequently, cellular polarity and adhesive capacity are compromised, which facilitates the infiltration of cancer cells into either blood vessels or lymphatic vessels and hence promotes distant metastasis. This phenomenon makes the cancer cells more aggressive and migratory (Pastushenko and Blanpain, 2019). In a study conducted by (Zhou et al., 2021), it was revealed that vitexin can increase the expression of E-cadherin. By strengthening the expression of this protein, vitexin effectively weakens the ability of gastric cancer cells to undergo EMT. This finding emphasizes the potential of vitexin as a therapeutic component for reducing the invasive potential of cancer cells and limiting the occurrence of distant metastasis.

#### 4.5.4 Regulation of TME

TME is composed of inflammatory, hypoxic, and immune microenvironments, creating an ideal environment for cancer cell proliferation and viability (Hu et al., 2022). Within the hypoxic TME, hypoxia-inducible factor (HIF) is highly expressed (Jiang et al., 2020), which directly induces the transcription of angiogenic factors, promoting tumor angiogenesis. Studies have shown that hawthorn’s ethanol extract can reduce HIF-1α activity in hypoxia-induced prostate cancer cells. Additionally, it inhibits hypoxia-induced angiogenesis and impacts the growth of cancer cells by regulating target genes associated with various aspects of cancer progression (Lee et al., 2017).

### 4.6 Induction of ROS produced

ROS, which are products of oxygen consumption or cell metabolism, have the ability to regulate the development and survival of cancer by influencing the tumor environment and tumor matrix ingredients (Cheung and Vousden, 2022). ROS at different concentrations play distinct roles in cancer cells. Low concentrations promote cancer occurrence, while high concentrations increase oxidative damage to cancer cells, potentially leading to their death through ROS-dependent death signals (Liang R. S. et al., 2023). According to current beliefs, the buildup of intracellular ROS has the potential to trigger different types of cancer cell death (Villalpando-Rodriguez and Gibson, 2021). As a result, controlling the level of intracellular ROS has emerged as a promising and effective strategy for treating cancer.

ROS is a well-known apoptosis-stimulating factor (Pant et al., 2017). Current studies have demonstrated that certain anticancer active ingredients found in hawthorn, such as isoorientin (Xu et al., 2020), isorhamnetin (Li Y. et al., 2022), and ursolic acid (Kang et al., 2022), can play a role through the ROS-mediated mitochondrial-dependent apoptosis pathway. It is mainly manifested in the induction of ROS production after drug treatment. The significant accumulation of ROS disrupts the integrity and function of mitochondria, ultimately activating caspase-3 or caspase-9 and initiating apoptosis (Tu et al., 2021). Additionally, ROS production triggers endoplasmic reticulum stress, which results in the accumulation of Ca^2+^ and the induction of apoptosis in cancer cells. Stigmasterol (Bae et al., 2020) and β-sitosterol (Bae et al., 2021) can enhance ROS production and endoplasmic reticulum stress through the endoplasmic reticulum-mitochondrial axis, thereby triggering pro-apoptotic signals and exerting anticancer effects.

### 4.7 Inhibition of inflammatory molecule production involved in the carcinogenesis process

Earlier research has established a significant link between inflammation and the creation of cancer cells, suggesting that specific inflammatory reactions may contribute to the development of cancer (Mantovani et al., 2008). Consequently, targeting molecules that inhibit inflammation and participate in inflammatory processes may be a good cancer prevention and treatment strategy. Recent research on orientin has demonstrated its ability to decrease cell viability, lower the expression of inflammatory cytokines, and suppress the production of inflammatory mediators, ultimately impeding the advancement of cancer (Tian et al., 2019).

## 5 Regulation of hawthorn on anticancer signaling pathways

**FIGURE 4:**
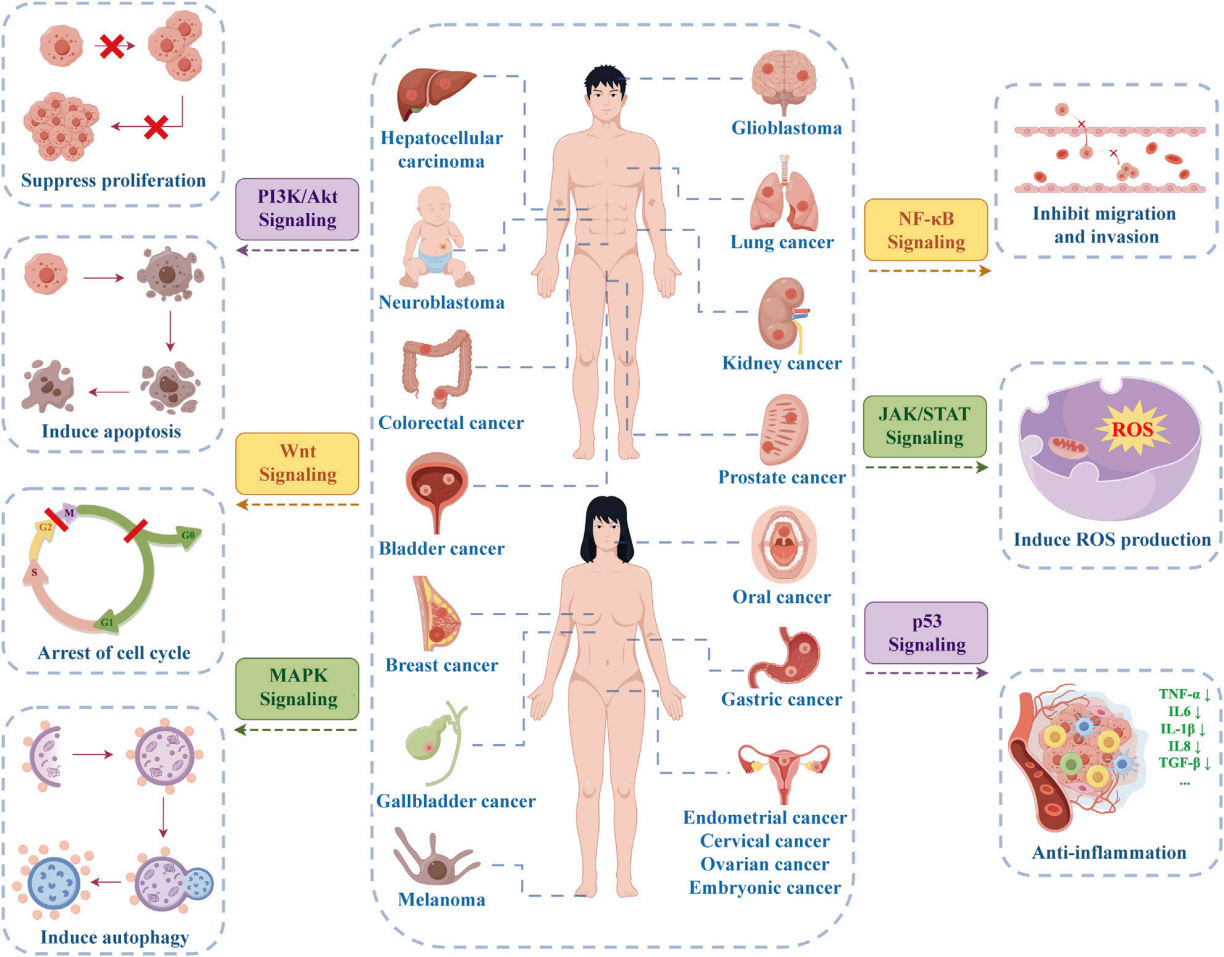
The anticancer mechanisms and action pathways of hawthorn (By Figdraw, http://www.figdraw.com/).

It is evident that the abnormal activation of certain signaling pathways can impede the apoptosis and autophagy of cancer cells, promote their proliferation, facilitate the cell cycle process, enhance invasion and metastasis, bolster drug resistance, and consequently drive cancer development or progression (Sanchez-Vega et al., 2018). Extracts and anticancer active ingredients of hawthorn can effectively treat cancer by regulating these signaling pathways. The anticancer mechanisms and signaling pathways of hawthorn are illustrated in Figure 4.

### 5.1 PI3K/Akt signaling pathway

The PI3K/Akt pathway is a well-known signaling pathway implicated in cancer (Murugan, 2019). An experiment has provided evidence that vitexin can promote apoptosis in A549 cells via the PI3K/Akt/mTOR pathway. This process coincides with a reduction in the expression levels of p-PI3K, p-Akt, and p-mTOR, implying that vitexin possesses therapeutic potential in NSCLC (Liu et al., 2019). Similarly, hyperoside has been discovered to induce cell cycle arrest in the G1 phase by inhibiting this pathway in studies involving hepatocellular carcinoma (Wei et al., 2021). Additionally, isorhamnetin has demonstrated its ability to induce apoptosis and impede gallbladder cancer cells cycle through this pathway, providing new avenues for cancer treatment (Zhai et al., 2021).

### 5.2 Wnt signaling pathway

Cancer initiation, maintenance, and progression can be linked to abnormal Wnt signaling (Zhang and Wang, 2020). This signaling pathway encompasses two major parts: the classic Wnt/β-catenin pathway and the non-canonical Wnt pathway. The latter operates independently of β-catenin’s transcriptional activity (Semenov et al., 2007).

A study on the methanol extract of hawthorn berries revealed its potential for inhibiting breast cancer cell growth and arresting the cell cycle at the G1/S phase through the regulation of the Wnt signaling pathway (Kombiyil and Sivasithamparam, 2023). Furthermore, hawthorn polysaccharide extract may hinder the activation of the Wnt/β-catenin signaling pathway by upregulating miR-146a-5p levels, resulting in the suppression of AGS gastric cancer cell proliferation and induction of apoptosis (Li X. P. et al., 2021). Additionally, it has been discovered that ursolic acid can impede the malignant phenotype of colorectal cancer and interrupt the cell cycle by mitigating the Wnt/β-catenin signaling axis (Zhao et al., 2023).

### 5.3 MAPK signaling pathway

This signaling pathway can regulate a variety of cellular mechanisms, thereby affecting biological processes. In the context of cancer, this pathway is particularly significant as it affects cancer proliferation, apoptosis, invasion, and metastasis (Peluso et al., 2019).

Scientific investigations have demonstrated that hawthorn acid, a notable constituent of hawthorn, possesses the capacity to alter the levels of ROS in malignant cells, ultimately resulting in programmed cell death (Jain and Grover, 2020). Moreover, it can repress the activity of proteins linked to the MAPK/ERK signaling pathway to hinder cell migration and invasion. These findings emphasize its potential as a promising drug for combating cancer (Liu et al., 2020). In a separate study on the effects of isorhamnetin on oral squamous cell carcinoma cells, it was discovered that isorhamnetin has the capability to halt the cell cycle during the G2/M phase and instigate apoptosis via ROS and ERK/MAPK pathways (Chen et al., 2021).

### 5.4 NF-κB signaling pathway

NF-κB is a crucial inducible transcription factor that plays a significant role in cell proliferation, differentiation, apoptosis, and carcinogenesis. Upon receiving internal and external stimuli, cells activate this signaling pathway, which results in the binding of nuclear factors to specific genes and the subsequent regulation of target gene expression (Chauhan et al., 2022; Deka and Li, 2023).

Frequently, the stimulation of this signaling cascade is accompanied by the emergence of cancer or inflammation (Taniguchi and Karin, 2018). In an investigation carried out by Tao J. Y. et al. (2023), it was elucidated that the activation of the NF-κB signaling pathway can be impeded by orientin and curb the proliferation and migration of cancer cells *in vitro*. Similarly, Wang L. et al. (2020) detected that chlorogenic acid can hinder the activation of this signaling pathway and the expression of downstream anti-apoptotic genes in cells, displaying a noteworthy inhibitory impact on the proliferation of lung adenocarcinoma cells.

### 5.5 Janus kinase (JAK)/STAT signaling pathway

The speedy transmission of signals from the cell membrane to the nucleus is facilitated by this pathway, which regulates the expression of downstream target genes through the activation of STAT and plays a crucial role in governing cancer cell proliferation and metastasis (Xue C. et al., 2023). STAT3, a key member of the STAT protein family, and its excessive activation contribute to the promotion of malignant biological behavior (Huynh et al., 2019; Zou et al., 2020).

By investigating the impact of vitexin on the STAT3 signaling pathway and key cancer markers, researchers have demonstrated that vitexin can successfully disrupt the sustained activation of JAK1, JAK2, and STAT3 in hepatocellular carcinoma (HCC) cells. These findings suggest that vitexin may serve as a potent inhibitor of the STAT3 pathway, offering the potential to suppress the proliferation and invasion of HCC cells (Lee et al., 2020).

### 5.6 p53 signaling pathway

p53, a well-researched suppressor of tumor growth, plays a crucial role in initiating various biological responses. Its abnormal activation has a strong connection to the onset and progression of cancer (Huang, 2021; Shen et al., 2023). Yang et al. (2013) observed an upregulation of p53 expression and its downstream genes, p21^WAF1^ and Bax, upon exposure of oral cancer cells to vitexin. When p53 activity was suppressed, vitexin lost its anticancer effect, suggesting that vitexin can induce apoptosis through the p53-dependent pathway.

## 6 Combination therapy in cancer treatment to enhance efficacy and reduce toxicity

Cancer poses a significant threat to human health due to its high mortality rate, highlighting the ongoing challenges in cancer research. Drug therapy is a crucial component in cancer treatment, but the toxic side effects and drug resistance of chemical drugs, such as chemotherapeutics, limit their effectiveness (Hussain et al., 2021). TCM extracts and their active ingredients have been recognized as natural anti-tumor agents. In recent years, the integration of natural compounds with chemical drugs has emerged as a prominent approach in cancer therapy, leveraging their distinctive complementary benefits. This combined therapy not only efficiently hinders cancer advancement and enhances the effectiveness of chemical drugs but also mitigates adverse effects and facilitates drug resensitization (Wang et al., 2018; Wang X. et al., 2023).

Research has demonstrated that combining hawthorn extracts and key anticancer compounds like ursolic acid, hyperoside, and maslinic acid, along with chemical drugs, can effectively amplify the mentioned benefits. Thus, comprehending the mechanisms underlying various combination therapy approaches holds positive significance in refining cancer treatment modalities and enhancing patients’ quality of life.

### 6.1 Study on the enhancing effect of combination drugs

#### 6.1.1 Enhance the anticancer efficacy of chemical drugs

The combination of hawthorn extract and its active ingredients with chemotherapy drugs can target tumor tissues through multiple targets and pathways, enhancing the anti-tumor effectiveness of chemotherapy drugs by regulating various signal transduction pathways. Doxorubicin (DOX) is a highly effective chemotherapy drug in clinical practice (Cortés-Funes and Coronado, 2007). When ursolic acid is combined with DOX, it inhibits the proliferation and migration of highly invasive cells while also enhancing programmed cell death and promoting late apoptosis of cancer cells. The mechanism of action may involve targeting the PI3K/Akt signaling pathway and activating the Hippo signaling pathway (Hu et al., 2023). The combined treatment of maslinic acid and cisplatin demonstrated a more potent inhibitory effect compared to either drug alone. This enhanced effect was attributed to the downregulation of XIAP and other genes, leading to a decrease in caspase inhibition, increased levels of active caspases, and ultimately higher rates of apoptosis in lung cancer cells (Bai et al., 2017). Similarly, the co-administration of hawthorn leaf extract with cisplatin was found to enhance the efficacy of low-dose cisplatin, resulting in a significant reduction in cancer cell viability. These findings suggest that the combination therapy of these compounds could be a promising approach for lung cancer treatment (Omairi et al., 2020). The studies mentioned above offer compelling evidence supporting the use of active components and extracts from hawthorn as potential novel anticancer strategies and adjunctive chemotherapy agents.

In addition to the synergistic effect of a single chemotherapeutic drug, hawthorn’s active ingredients can also be combined with chemotherapeutic and targeted drugs (Nishimoto, 2022). Research has shown that vitexin can potentially block the STAT3 signaling cascade, specifically inhibiting STAT3 activity. When combined with the chemotherapeutic drug DOX and the multi-target antitumor drug sorafenib, it demonstrates significant pro-apoptotic and anti-invasive effects, indirectly hindering tumor growth and progression, and demonstrating potential for enhancing efficacy (Lee et al., 2020).

The use of non-steroidal anti-inflammatory drugs (NSAIDs) as potential cancer preventers is also a novel approach in anticancer strategies. NSAIDs, such as aspirin, can suppress the expression of prostaglandin-endoperoxide synthase 2 (COX-2) by inhibiting NFKB1 activity, thereby reducing DNA damage and impeding cancer progression (Maniewska and Jeżewska, 2021). Recent studies have shown that the combined administration of vitexin and aspirin inhibits colorectal cancer cell growth, induces apoptosis, reduces invasion capabilities, and suppresses inflammation-related factors, including COX-2. These findings suggest that the synergistic effect of using both compounds together is highly effective (Chen D. et al., 2023).

#### 6.1.2 Enhance the sensitivity of chemotherapy drugs

The combination of Chinese and Western medicine can not only enhance the efficacy of chemotherapy drugs, but also increase the sensitivity of cancer cells to these drugs. Current research indicates that traditional DOX-based chemotherapy regimens face limitations due to drug resistance and other factors. Therefore, utilizing natural compounds found in hawthorn as an adjunct treatment to restore cancer cells’ sensitivity to DOX holds significant promise in cancer therapy. Recent findings by Yang T. et al. (2023) demonstrate that isorhamnetin alone exhibits moderate cytotoxicity towards DOX-resistant cancer cells, leading to cell cycle arrest and activation of the apoptotic pathway. When used in conjunction with DOX, isorhamnetin significantly inhibits tumor growth and reduces tumor burden in mouse models compared to DOX treatment alone. Thus, isorhamnetin can serve as a sensitizer for DOX to enhance its therapeutic efficacy. When hyperoside is combined with paclitaxel, it has been demonstrated to enhance the efficacy of paclitaxel in treating breast cancer. This combination indirectly boosts the toxicity of paclitaxel towards breast cancer cells and induces apoptosis by inhibiting the toll-like receptor 4 (TLR4)-mediated pro-inflammatory and pro-survival pathways. The study successfully reduced paclitaxel resistance and increased the sensitivity of breast cancer cells to paclitaxel through combination therapy, offering a promising new treatment approach for breast cancer patients (Sun et al., 2020). Additionally, the combination of procyanidin B2 and docetaxel (DOCE) exhibited an adjuvant therapeutic effect. Compared to treatment with DOCE alone, the combined therapy increased the pro-apoptotic effect by 2–5 times. Procyanidin B2 enhances the efficacy of chemotherapeutic drugs through a sensitization mechanism, effectively mitigating drug resistance and adverse reactions (Núñez-Iglesias et al., 2021).

#### 6.1.3 Reverse the resistance of cancer cells to chemotherapy drugs

Drug resistance in cancer cells is a significant factor contributing to treatment failure and disease recurrence. Multidrug resistance (MDR) stemming from chemotherapy resistance is a major hurdle to the effectiveness of anticancer drugs (Sarmento-Ribeiro et al., 2019; Wang J. Q. et al., 2021). Hence, the development of high-efficiency and low-toxicity reversal agents is crucial for combating tumor resistance and maximizing the potential of anticancer drug therapy. TCM has demonstrated promising applications in this area (Chen T. et al., 2024). The combination of chemotherapeutic drugs and active ingredients found in hawthorn has displayed distinct advantages in effectively reversing tumor drug resistance.

Currently, some major chemotherapy drugs like cisplatin, DOCE, and DOX have been reported to exhibit resistance, with complex resistance mechanisms (Kar et al., 2024). There is growing evidence supporting ferroptosis as a significant mechanism of chemotherapy resistance. Bioactive compounds acting as ferroptosis inducers, either alone or in combination with other chemotherapeutic drugs, can trigger cancer cell death, particularly in drug-resistant cancer cells, thereby overcoming resistance (Li B. et al., 2020; Wang Y. et al., 2023). The combined use of isoorientin and cisplatin has been shown to induce ferroptosis, significantly reducing the viability of drug-resistant cancer cells and increasing sensitivity to cisplatin. *In vivo* experiments have further indicated that isoorientin enhances the concentration of cisplatin in tumor cells, reversing drug resistance and enhancing cisplatin’s efficacy (Feng et al., 2023).

Reducing the overexpression of ATP-binding cassette transporter proteins is also a crucial strategy to combat MDR (Wu et al., 2023). Increased expression of these proteins can result in the efflux of anti-tumor drugs from cancer cells, leading to decreased intracellular drug concentrations below effective levels and ultimately causing cell resistance. One extensively studied ATP-binding cassette transporter protein is P-glycoprotein/ABCB1 (Engle and Kumar, 2022), which can be inhibited by maslinic acid to reverse resistance to DOCE. Maslinic acid functions by initially inhibiting the transcriptional activity of the proliferation-related transcription factor forkhead box protein M1 (FoxM1), subsequently reducing the expression of the downstream target ABCB1. This inhibition prevents drug efflux, allowing for the accumulation of DOCE within cancer cells and thereby restoring their responsiveness to DOCE (Wang K. et al., 2020). Gawel et al. (2019) discovered that MIX2, a natural extract mixture of fresh fruits containing hawthorn, can modulate the expression of the drug efflux protein P-glycoprotein, leading to enhanced sensitivity of cancer cells to DOX. Consequently, concurrent exposure to MIX2 and DOX can induce cell death in cancer cells that were initially resistant to DOX. This highlights the potential of MIX2 as a promising candidate for overcoming drug resistance in malignant tumors.

At the same time, cancer cells have the ability to reduce DNA damage by activating repair mechanisms, leading to drug resistance (Bouwman and Jonkers, 2012). Research indicates that 5-FU can disrupt the synthesis of DNA and RNA by blocking thymidine synthase, ultimately triggering apoptosis (Yang et al., 2021). Luo and Liu (2021) discovered that maslinic acid can partially reverse cancer cells’ resistance to 5-FU. The combined use of maslinic acid and 5-FU not only enhances the anticancer efficacy but also reduces the individual dose of 5-FU appropriately. Analysis of DNA damage repair marker proteins indicated that both 5-FU and maslinic acid can independently suppress the expression of these proteins in drug-resistant cells, with a more significant impact observed when used in combination. Maslinic acid’s ability to reverse drug resistance seems to be associated with its inhibition of DNA damage repair mechanisms in cancer cells.

### 6.2 Study on reducing toxicity of combination drugs

#### 6.2.1 Reduce the damage of chemotherapeutic drugs to normal tissues and organs

Chemotherapy is a systemic treatment that not only targets cancer cells but also has toxic and side effects on normal tissues and organs. These effects primarily impact the nervous system, hematopoietic system, liver and kidney function, digestive system, reproductive system, and heart (Fu et al., 2018). Therefore, the utilization of TCM as an adjuvant to chemotherapy is crucial to mitigate the harm to the body caused by chemotherapy, ultimately enhancing clinical outcomes and the quality of life for patients (Zhang et al., 2018).

The n-butanol extract of hawthorn was analyzed chemically by researchers, revealing that polyphenols and flavonoids exhibited strong antioxidant properties. These compounds were able to counteract free radicals, reduce oxidative stress induced by DOX, thus mitigating its toxicity and safeguarding the heart, kidneys, and liver from harm (Mechri et al., 2018). Since DOX-induced reproductive toxicity is also linked to oxidative stress, the antioxidant properties of hawthorn water extract demonstrate a protective effect against reproductive toxicity during DOX treatment. Combining hawthorn aqueous extract with DOX substantially alleviates oxidative damage to testicular tissue associated with DOX through antioxidant reactions, facilitating the restoration of testicular tissue morphology and enhancement of semen quality (Shalizar Jalali and Hasanzadeh, 2013). Isoorientin can enhance the survival rate of DOX-injured cardiomyocytes by mitigating oxidative stress, reducing mitochondrial dysfunction, and inhibiting apoptosis. This leads to a protective effect on myocardial tissue and a decrease in cardiotoxicity. In a study by Li S. et al. (2022), it was observed that the combination of isoorientin and DOX resulted in a gradual return to normal myocardial morphology and improved survival status in mice undergoing DOX chemotherapy. Additionally, chlorogenic acid has been shown to alleviate histological abnormalities and peripheral neuropathy induced by cisplatin, demonstrating a protective effect against neurotoxicity *in vitro*, as reported by Ünel et al. (2023).

#### 6.2.2 Reduce the serious adverse effects of cancer

Cancer cachexia, cancer-related fatigue, and cancer pain are prevalent clinical syndromes in patients with advanced cancer. These adverse reactions directly impact cancer therapy outcomes, diminish patients’ quality of life, and may even influence prognosis (Baracos et al., 2018; Thong et al., 2020; Mercadante et al., 2024). Cancer cachexia, characterized by reduced adipose tissue and skeletal muscle consumption, is a metabolic disorder resulting from tumor and body factors (Argilés et al., 2023). Despite the lack of approved treatments (Biswas and Acharyya, 2020), studies have shown that ursolic acid, a compound found in hawthorn, exhibits promising therapeutic potential for this condition. Administering ursolic acid in the advanced stages of tumor growth has been found to delay muscle atrophy, mitigate cancer cachexia progression by activating SIRT1 and inhibiting NF-κB and STAT3 pathways, decrease inflammatory cytokine levels in cancer mice (Tao W. et al., 2023), enhance food intake, and prevent weight loss (Chen L. et al., 2024). In summary, the synergistic effect of combining hawthorn with chemical drugs can enhance efficacy, reduce toxicity, and offer additional treatment options for malignant tumors.

## 7 Novel drug delivery systems

Hawthorn extract and its main anticancer active ingredients face challenges in terms of low bioavailability and limited targeting of diseased tissue, which greatly hinder their development and application (Qiu et al., 2023). Compared to traditional preparations, novel drug delivery systems offer several advantages. They allow for better control of drug release, prolong drug action time, and increase drug concentration at the site of the lesion, thereby improving bioavailability, enhancing targeting, reducing potential side effects, and improving overall efficacy (Qiao et al., 2020). At present, novel drug delivery systems for anticancer drugs, including liposomes, microspheres, nanoparticles, microemulsions, cyclodextrins, polymer micelles, *etc.*, have achieved great success in delivering natural bioactive ingredients (Ke et al., 2022; Zhang Y. L. et al., 2023). In fact, these techniques have also proven successful in delivering the active ingredients of hawthorn.

Selenium, an essential trace element for the human body, has been proven to have significant anticancer effects (Xiao et al., 2023). However, there is a narrow range between the effective dose and the toxic dose of selenium. To overcome this limitation, researchers have explored the combination of nanotechnology and selenium, which not only enhances the anticancer effects but also widens the application window of selenium while reducing its toxicity (Ferro et al., 2021). Building upon this advantage, Cui et al. (2018) utilized hawthorn fruit extract (HE) as a reducing agent to create HE-selenium nanoparticles. These nanoparticles possess the ability to induce oxidative stress, disrupt mitochondrial function, and trigger apoptosis in HepG2 liver cancer cells via the mitochondrial pathway.

Gold and silver nanoparticles have demonstrated promising anticancer properties and low toxicity to humans (Xu J. J. et al., 2022). However, the synthesis of these nanoparticles may result in the production of harmful substances that could endanger human health and the environment (Rai and Yadav, 2013). To address this issue, researchers utilized the aqueous extract of hawthorn leaves as both a reducing agent and protective agent in the synthesis of precious metal nanoparticles CML@X-NPs. Cytotoxicity experiments conducted on gastric adenocarcinoma and breast cancer cell lines revealed a significant anticancer effect of CML@X-NPs. This discovery offers valuable insights for the development of safer and more environmentally friendly nanoparticles, as well as their potential application in cancer treatment (Shirzadi-Ahodashti et al., 2020).

Microspheres, composed of high-molecular-weight polymers, are spherical or quasi-spherical entities used as carriers for drug encapsulation. These small particles have the ability to penetrate the biofilm barrier, enabling targeted drug release in specific areas (Ayyanaar and Kesavan, 2023). One commonly used method for drug inclusion is cyclodextrin inclusion technology, with β-cyclodextrin being a popular choice due to its low biological toxicity and good biocompatibility (Boroushaki and Dekamin, 2023). It also improves drug delivery efficiency (Sheng and Kumar, 2022). Ding et al. (2023) successfully prepared vitexin inclusion complex microspheres using β-cyclodextrin. This approach effectively addressed the challenges of vitexin’s insolubility and low bioavailability *in vivo*. Furthermore, the microspheres exhibited promising anti-colorectal cancer effects by inducing apoptosis and inhibiting cell proliferation.

Liposomes are microvesicles composed of lipid bimolecules arranged in a specific manner, which can enhance the accumulation of drugs in target tissues and provide sustained release effects (Fernandes, 2023). In light of the rapid progression of glioma and the limited effectiveness of current clinical treatments (Cao et al., 2023), developed a device to facilitate the quick and effective synthesis of vitexin/indocyanine green liposomes. The evaluation of vitexin release from these liposomes *in vitro* demonstrated strong inhibitory effects on glioma cell proliferation and migration. Furthermore, liposomes are characterized by their small and uniform particle size distribution, which effectively enhances cumulative release *in vitro*, improves the solubility of poorly soluble drugs, and enhances the anticancer effect.

Solid lipid nanoparticles (SLNs) have gained extensive use in nanotechnology-based drug delivery systems, playing a crucial role in clinical medicine, cancer treatment, and other domains (German-Cortés et al., 2023). The primary purpose of SLNs is to improve the stability of enclosed drugs and facilitate targeted drug release (Sivadasan et al., 2023). Research has demonstrated that SLNs have been successful in increasing the solubility of maslinic acid, resulting in a substantial influence on the preciseness and efficacy of targeted diagnosis and treatment. This makes SLNs a promising option as a drug carrier for delivering maslinic acid (Aguilera-Garrido et al., 2023).

## 8 Clinical trials

The ultimate goal of theoretical and basic research on TCM is to successfully apply effective drugs to the market, with clinical trials serving as the pivotal step towards this objective. The establishment of clinical trials for TCM is vital in advancing its modernization and international recognition and has a positive impact on improving the scientific validity, safety, and efficiency of TCM (Sun et al., 2021).

With these goals in mind, we conducted searches using the keywords “hawthorn” and “cancer” to identify relevant clinical trials both domestically and internationally on the International Clinical Trials Registry Platform (https://trialsearch.who.int/). The search results indicated that the hawthorn red pigment could potentially serve as an adjuvant therapy drug when combined with standard analgesics for managing cancer pain (Phase Ⅰa Clinical Study of Hawthorn Red Pigment Combined With Standard Analgesic for Refractory Cancer Pain, trial registration number: NCT05561023). This trial is significant in investigating the potential toxicity-reducing effects of hawthorn combination therapy in cancer treatment. The use of hawthorn in managing cancer pain may offer a more cost-effective, efficient, and less side-effect approach (Wang et al., 2018).

However, currently, there is a notable absence of large-scale, rigorously designed and implemented clinical trials to conclusively establish the anticancer properties of hawthorn. Although network pharmacology and *in vitro* and *in vivo* experiments have confirmed the anticancer potential of hawthorn, suggesting that it could be used as adjuvant therapy in cancer treatment, further validation of hawthorn’s efficacy in combating cancer through clinical trial data is required, given the complexity of cancer treatment and the unique nature of TCM. Only through clinical trials can the optimal dose and mode of administration of the drug be determined, as well as comprehensively assessing its side effects and potential risks (Ness and Royce, 2017; Cabibbo et al., 2024). Furthermore, clinical trials can provide further clarification on the pharmacological mechanism and systematically verify the anticancer efficacy of hawthorn using scientific methods. This rigorous process ensures that drug data is thoroughly validated and meets both research and regulatory standards (Lewis et al., 2016; Choudhari et al., 2019). Therefore, clinical trials investigating the anticancer effects of hawthorn are crucial for drug development and innovation, representing the only pathway to establish hawthorn as a new cancer treatment option. This perspective undoubtedly offers new insights and momentum for future research on hawthorn.

## 9 The health value of hawthorn: cancer prevention

In the realm of cancer treatment, TCM offers a comprehensive approach to prevention and control throughout the entire process. This approach includes early prevention, postoperative recurrence prevention, reduction of chemotherapy toxicity and enhancement of efficacy, as well as prevention and treatment of tumor complications (Liu et al., 2022). Cancer development is a chronic and progressive process, requiring prolonged intervention for prevention. Utilizing phytochemicals from medicinal and edible homologous herbs to provide essential nutrients, along with harnessing functional factors for cancer prevention and adjunctive therapy, can effectively eliminate precancerous factors, reduce cancer risk, achieve early cancer prevention, and contribute to overall health maintenance (Montégut et al., 2022).

In recent years, the application of hawthorn has gradually aligned with the trend of cancer prevention. Using “hawthorn” as a search term, we conducted a search on the special food query platform (http://ypzsx.gsxt.gov.cn/specialfood/#/food) of China’s State Administration of Market Regulation and discovered that health products predominantly formulated with hawthorn in combination with other traditional Chinese medicinal ingredients possessing both nutritional and health-promoting functions, while also showcasing the potential for cancer prevention. This is specifically evident in three aspects: first, enhancing immunity to maintain or improve overall health (Yang et al., 2024); Second, assisting in protecting gastric mucosa, inhibiting damage to gastric mucosa, and reducing the risk of gastric cancer development (Wang H. et al., 2020; Păun et al., 2020; Xu W. et al., 2022; Yi et al., 2022; Zhong et al., 2023); Third, aiding in protecting against chemical-induced liver damage, reducing the hepatotoxicity of chemical products, and thereby decreasing the incidence of liver cancer (Han et al., 2020; Atia et al., 2022). Relevant health products are illustrated in Table 1, indicating that strengthening the application of such health products has a positive effect on cancer prevention.

**TABLE 1:** Health products related to hawthorn.

Efficiency	Product name	Principal raw material	License number
Boost immunity	Maizhibao brand Gingo leaf Ganoderma lucidum Hawthorn capsule	Cordyceps militaris powder (irradiated), Green tea extract, Hawthorn extract, Ginkgo biloba extract, *Gynostemma pentaphyllum* extract, Lycopene powder, Ganoderma lucidum extract	G20170071
	Meishantang brand Ganoderma lucidum Hawthorn Ganoderma lucidum spore powder capsule	Ganoderma lucidum, Hawthorn, Broken Ganoderma lucidum spore powder (irradiated)	G20130753
	Bangruite brand Astragalus Hawthorn Prince Ginseng tablets	Astragalus extract, Hawthorn extract, Radix Pseudostellariae extract, Poria extract, Atractylodes extract	G20140373
Auxiliary protection from chemical liver injury	Deni brand Hawthorn Salvia Plum capsule	Hawthorn extract, Plum extract, Salvia extract	G20141182
	Baobei brand Gynostemma Pueraria Hawthorn granules	Gynostemma, Pueraria, Hawthorn, Phyllanthus emblica, Schisandra	G20160116
	Langlangshangkou brand Hawthorn Salvia drinks	Hawthorn, Salvia, Oyster, Zelan, Dandelion, Citron, Mulberry leaves	G20150579
Auxiliary protection from gastric mucosal injury	Hengxin brand Hericium erinaceus Hawthorn probiotic powder	Isomaltooligosaccharid, Hericium erinaceus extract, Hawthorn extract, *Lactobacillus* acidophilus powder, *Lactobacillus* casei powder, *Streptococcus* thermophilus powder, Bifidobacterium bifidum powder	G20150080

## 10 Conclusion and future perspective

Cancer is a public health problem of common concern in the world. At present, the prevention and treatment of cancer pose a heavy burden, necessitating the exploration of more effective and safe anticancer drugs. Numerous studies have demonstrated that TCM extracts and their active ingredients have shown promise in cancer treatment through various mechanisms. The medicinal and edible Chinese medicine hawthorn, containing vitexin, isoorientin, epicatechin, proanthocyanidins, and maslinic acid, has demonstrated anticancer activity both *in vivo* and *in vitro*. In light of this, we conducted a comprehensive review of relevant literature to examine the recent research progress on the mechanisms and pathways associated with hawthorn’s anticancer activity. The aim is to provide a foundation for future research and the development of new drugs derived from hawthorn.

Through literature retrieval, four reviews have been found that discuss the pharmacological and phytochemical effects of hawthorn (Zhang L. L. et al., 2020; Martinelli et al., 2021; Li R. et al., 2023; Cui et al., 2024). These reviews primarily focus on summarizing the botanical description and distribution, traditional usage, chemical composition, and pharmacological effects of hawthorn. Li R. et al. (2023) provided a comprehensive description of hawthorn but only briefly mentioned its anticancer potential. Building upon this foundation, our review explores the anticancer potential of hawthorn based on relevant literature and network pharmacology, while also predicting anticancer-related targets. We summarize the active ingredients of hawthorn and provide a systematic description of the anticancer mechanisms and associated signaling pathways. We also discuss the benefits of combining hawthorn with anticancer drugs to reduce toxicity and enhance efficacy, as well as the use of a novel drug delivery system incorporating hawthorn and various materials for tumor suppression. Additionally, we address the current limitations and future directions of clinical trials involving hawthorn and cancer. Finally, we emphasize the medicinal and dietary characteristics of hawthorn, highlighting its role in cancer prevention as a health food and underscoring its significance in anticancer research.

Based on our review, it is evident that there is still room for advancement in the scientific research of hawthorn. The chemical composition of hawthorn is intricate, and while current studies have extensively examined and reported on the bioactive substances responsible with anticancer properties, the current evaluation systems often lack accuracy in assessing its anticancer function. Therefore, it is crucial to include key pharmacodynamic substances such as vitexin, hyperoside, proanthocyanidins, ursolic acid, maslinic acid, *etc.*, and establish a scientifically sound method for evaluating the efficacy of hawthorn. Secondly, several recent experimental studies have demonstrated the anticancer properties of hawthorn. Hawthorn extracts and its main active ingredients have shown anticancer effects through various mechanisms. These effects are achieved by regulating signaling pathways such as PI3K/Akt, MAPK, and Wnt/β-catenin. However, it is worth noting that the majority of research findings are based on *in vivo* and *in vitro* experiments, with limited attention given to clinical trials. Therefore, to fully confirm and harness the anticancer potential and clinical efficacy of hawthorn, it is essential to gradually conduct clinical trials alongside the existing experimental research. Thirdly, the low bioavailability of orally administered hawthorn-related compounds can be attributed to their hydrophobicity and first-pass metabolism. Recent investigations focusing on novel drug delivery systems have exhibited encouraging outcomes in terms of enhancing bioavailability, improving targeting capabilities, and augmenting the efficacy of tumor suppressors in hawthorn utilization. However, these studies are limited and lack clinical data support. Therefore, future research should focus on larger-scale and more in-depth investigations to promote the development and clinical application of new hawthorn formulations. Besides that, current research on the combination of hawthorn primarily focuses on increasing efficacy, with limited studies on the mechanism of reducing toxicity. Areas such as cancer pain, cancer-related fatigue, and chemotherapy-induced gastrointestinal side effects, including loss of appetite, nausea, and vomiting, warrant further exploration and analysis.

## Figures and Tables

**Table:** 

**AMPK**	Adenosine monophosphate-activated protein kinase
**AP-1**	Activator protein-1
**ATG5**	Autophagy related 5 homolog
**Bax**	Bcl-2-associated X protein
**Bcl-2**	B cell lymphoma-2
**CA9**	Carbonic anhydrase IX
**CDK19**	Cyclin-dependent kinase 19
**CDKs**	Cyclin-dependent kinases
**C-MYC**	V-myc myelocytomatosis viral oncogene homolog
**COX-2**	Prostaglandin-endoperoxide synthase 2
**DOCE**	Docetaxel
**DOX**	Doxorubicin
**ECM**	Extracellular matrix
**EMT**	Epithelial-mesenchymal transition
**ERK**	Extracellular signal-regulated kinase
**FoxO1**	Forkhead box protein O1
**GSK-3β**	Glycogen synthase kinase 3β
**HCC**	Hepatocellular carcinoma
**HIF**	Hypoxia inducible factor
**HO-1**	Heme oxygenase-1
**IL–6**	Interleukin 6
**JAK**	Janus kinase
**LC3-II**	Microtubule-associated protein 1 light chain 3 II
**MAPK**	Mitogen-activated protein kinase
**MDR**	Multidrug resistance
**MMP-2**	Matrix metallopeptidase-2
**MMP-9**	Matrix metalloproteinase-9
**MMPs**	Matrix metalloproteinases
**mTOR**	Mechanistic target of rapamycin
**NF-κB**	Nuclear factor kappa-B
**NSAIDs**	Nonsteroidal anti-inflammatory drugs
**NSCLC**	Non-small cell lung cancer
**PI3K/Akt**	Phosphatidylinositol 3-kinase/Protein kinase B
**PKCα**	Protein kinase C alpha
**PPARγ**	Peroxisome proliferator-activated receptor γ
**PTEN**	Phosphatase and tensin homolog
**RORC**	Retinoic acid-related orphan receptor C
**ROS**	Reactive oxygen species
**SLNs**	Solid lipid nanoparticles
**STAT3**	Signal transducer and activator of transcription 3
**TCM**	Traditional Chinese medicine
**TLR4**	Toll-like receptor 4
**TME**	Tumor microenvironment
**VEGF**	Vascular endothelial growth factor
**VEGFA**	Vascular endothelial growth factor A
**Wnt**	Wingless/integrated
**YAP**	Yes-associated protein
