# Supplementary material for: Hawthorn with “homology of medicine and food”: a review of anticancer effects and mechanisms
Source: Front Pharmacol. 2024 Jun 10;15:1384189. doi: 10.3389/fphar.2024.1384189 (PMC11194443; doi:10.3389/fphar.2024.1384189)
Supplement: Supplementary file 1 [file Table1.docx]

Supplementary Material

# Supplementary Table

**Supplementary Table 1. The mechanism of hawthorn extracts and its main ingredients**

| **Tumor type** | **Component** | **In vivo / in vitro** | **Experimental model** | **Dose / IC_50_** | **Action mechanism** | **Ref** |
| --- | --- | --- | --- | --- | --- | --- |
| Cancer | New 8-O-4' neolignans | *In vitro* | Human cancer cell lines:  HeLa, HepG2, HT-1080, HCT116, MCF-7, A549, U-937, Mrc5, A375-S2, HL60, K562 | - | Cytotoxicity | ([Huang et al., 2013a](#_ENREF_15)) |
| Cancer | Dihydrobenzofuran neolignans | *In vitro* | Human cancer cell lines:  HeLa, HepG2, HT-1080, A375-S2, MCF-7, U-937, K562, HL60 | Compound 10 (HT-1080 cell line):  IC_50_=8.86 μM | Cytotoxicity | ([Huang et al., 2013b](#_ENREF_16)) |
| Cancer | Neolignans | *In vitro* | Human myeloma cells:  OPM2, RPMI-8226 | 50, 100 μmol/L | Cytotoxicity | ([Li et al., 2013a](#_ENREF_23)) |
| Gastric cancer | Methanolic and  acetone extracts (fresh leaves of *C. azarolus*) | *In vitro* | Human gastric adenocarcinoma cell line:  AGS | Methanolic extract (48h):  IC_50_=293.7 µg/mL  Acetone extract (48h):  IC_50_=576.6 µg/mL | ↓ Proliferaton | ([Mohammedsaeed and Mohamad, 2023](#_ENREF_32)) |
| Cancer | Methanolic extracts  (leaf of *C. pinnatifida*) | *In vitro* | Murine leukemia cell:  L1210;  Human cancer cells：  A549, SK-OV-3, SK-MEL-2, XF498, HCT15 | - | Cytotoxicity | ([Min et al., 2000](#_ENREF_31)) |
| Breast cancer | Petroleum ether, ethanol and water extracts (hawthorn stems and leaves) | *In vitro* | Human breast cancer cell lines:  MDA-MB-231, MCF7;  Non-tumorigenic cell line:  MCF 10A;  Peripheral blood mononuclear cell:  PBMC | MDA-MB-231:  IC_50_=43.9 mg/mL  MCF7:  IC_50_=47.6 mg/mL  MCF 10A:  IC_50_=45.3 mg/mL  PBMC:  IC_50_>75.0 mg/mL | Cytotoxicity;  ↓ Proliferaton | ([Maldonado-Cubas et al., 2020](#_ENREF_30)) |
| Human glioblastoma | Hawthorn leaf flavone | *In vitro* | Human astroblastoma cell：  U87 | 25, 50, 100 mg/L | ↓ Proliferation;  ↓ Migration;  ↓ Invasion | ([Diao et al., 2019](#_ENREF_6)) |
| Histiocytic lymphoma | Hawthorn leaf flavone | *In vitro* | Human histiocytic lymphoma cell:  U937 | 25, 50, 100, 150, 200 mg/L | ↓ Proliferaton | ([Tang et al., 2010](#_ENREF_46)) |
| Cancer | Phenolic extracts (buds and fruits of *C. monogyna*) | *In vitro* | Tumor cell lines:  MCF7, NCI-H460, HeLa, HepG2 | - | ↓ Proliferaton | **(**[Rodrigues et al., 2012](#_ENREF_41)**)** |
| Cancer | Triterpenoids (hawthorn berries) | *In vitro* | Human hepatocellular carcinoma cell:  HepG2;  Human breast cancer cell lines:  MDA-MB-231, MCF7 | ＜5 μM | ↓ Proliferaton;  ↓ Oxidation | ([Qiao et al., 2015](#_ENREF_39)) |
| Cancer | Corosolic acid;  Ursolic acid | *In vitro* | Cell lines: K-562, Hep G2, HeLa S3, A-549, SNU-C4 | Corosolic acid:  ED_50_=0.4-5.0 μg/mL;  Ursolic acid:  ED_50_=1.4-12.5 μg/mL | Cytotoxicity | ([Ahn et al., 1998](#_ENREF_1)) |
| Liver cancer | New lignan enantiomers (fruit of *C.pinnatifida*) | *In vitro* | Human hepatocellular carcinoma cell lines:  Hep3B, HepG2 | 1a (Hep3B):  IC_50_=34.97±2.74 μM;  1b (Hep3B):  IC_50_=17.42±0.71 μM | Cytotoxicity;  ↑ Apoptosis;  ↑ Autophagy | ([Shang et al., 2020](#_ENREF_42)) |
| Liver cancer | Racemic neolignans (fruit of *C.pinnatifida*) | *In vitro* | Human hepatocellular carcinoma cell lines:  Hep3B, HepG2 | 2a (Hep3B):  IC_50_=25.47 μM;  2b (Hep3B):  IC_50_=59.37 μM | Cytotoxicity;  ↑ Apoptosis | ([Guo et al., 2019a](#_ENREF_10)) |
| Human glioblastoma | Polyphenolic compounds (*Crataegus* berry, leaf, and flower extracts) | *In vitro* | Human glioblastoma cell line:  U87MG | 100, 250, 500 µg/mL | Cytotoxicity;  ↓ Proliferaton;  ↓ Invasive | (Żurek et al., 2021) |
| Hepatoma | Phenylpropanoids (fruit of *C.pinnatifida*) | *In vitro* | Human hepatocellular carcinoma cells:  Hep3B, HepG2 | 100 μM | Cytotoxicity;  ↑ Apoptosis | ([Guo et al., 2018](#_ENREF_9)) |
| Hepatoma | Phenylpropanoid and dibenzofuran derivatives (fruit of *C.pinnatifida*) | *In vitro* | Human hepatocellular carcinoma cell lines:  Hep3B, HepG2 | HepG2:  IC_50_=12.24 μM;  Hep3B:  IC_50_=24.9 μM | Cytotoxicity;  ↓ Proliferaton;  ↑ Apoptosis | ([Zhao et al., 2019](#_ENREF_61)) |
| Laryngeal cancer | Total flavonoids (hawthorn fruit) | *In vitro* | Human laryngeal cancer cell：  Hep-2 | - | ↓ Proliferaton | ([Zhang et al., 2004](#_ENREF_59)) |
| Skin tumor | Hot-water extracts  (dried fruit of *C. pinnatifida*) | *In vitro; In vivo* | Mouse epidermal cell line:  JB6 P+;  Female ICR mice  aged six weeks  (weight: 19.6±0.5 g) | 50, 100, 200 mg/mL | ↓ Inflammation;  ↓ AP-1 and NF-κB signals | ([Kao et al., 2007](#_ENREF_19)) |
| Melanoma | Ethyl acetate extract (leaves of *Crataegus azarolus*);  Vitexin-2''-O-rhamnoside | *In vitro* | Mouse melanoma cell line:  B16F10 | Ethyl acetate extract:  IC_50_=50 μg/mL;  vitexin-2''-O-rhamnoside:  IC_50_=20 μM | ↓ Proliferaton;  ↓ Tyrosinase activity | ([Mustapha et al., 2015](#_ENREF_33)) |
| Melanoma | Isolated compounds from hawthorn: total oligomer flavonoid (TOF);  (−)-Epicatechin | *In vitro; In vivo* | Mouse melanoma cell lines:  B16F10;  Primary human keratinocyte cells;  Male BALB/c mice  aged 6 and 8 weeks (weight: 20.0 ± 2.0 g) | TOF extract (48 h):  IC_50_=70 µg/mL;  EC (48 h):  IC_50_= 100 µmol/L;  150 mg/kg body weight for 21 days | ↓ Proliferaton;  ↓ Tyrosinase activity;  ↓ Oxidation;  ↑ Intracellular free radical scavenging activity | ([Mustapha et al., 2016a](#_ENREF_34)) |
| Laryngeal carcinoma | Polyphenol (water extracts of hawthorn) | *In vitro* | Human laryngeal carcinoma  cell:  HEp2 | 0.1–2.5× | Cytotoxicity | (Belščak-Cvitanović et al., 2014) |
| Colorectal cancer | Hawthorn water extracts | *In vitro* | Human colorectal adenocarcinoma cell：  Ca-2 | 0.5, 1, 2, 4 g/L | ↓ Proliferaton | ([Zhu, 2012](#_ENREF_64)) |
| Cancer | Extracts (fruits of *C. sanguinea*) | *In vitro; In vivo* | Kunming mice  Aged 6–8 weeks (weight: 18–22 g);  Fibroblasts of normal mouse:  3T3;  Human hepatoma cell line:  Q3;  Human laryngeal carcinoma cell line:  Hep-2;  Human gastric cancer cell line: MGC-803;  Human bladder carcinoma  cell line:  EJ;  Human lung cancer cell line:  SPC-A-1 | 1 g/mL, 100 mg/mL, 10 mg/mL, 1 mg/mL, 100 µg/mL | Cytotoxicity;  Antimutation | ([Sun et al., 2013](#_ENREF_43)) |
| Breast Cancer | Vitexin | *In vitro* | The human breast cancer cell line:  MCF-7 | 150 µM | ↓ Proliferaton;  ↑ let-7-b, let-7-c, AIB1;  ↓ miRNA-17-5p;  ↑ Apoptosis | ([Najafipour et al., 2022](#_ENREF_36)) |
| Cervical cancer | Hyperoside | *In vitro* | The human cervical cancer cell lines:  HeLa, C-33A | C-33A:  IC_50_=2 mM;  HeLa:  IC_50_=4 mM | ↓Proliferation;  ↓ C-MYC | ([Guo et al., 2019c](#_ENREF_12)) |
| Non-small cell lung cancer | Hyperoside | *In vitro; In vivo* | Adenocarcinoma lung cancer cell line:  PC-9;  T790M-positive NSCLC cell line:  NCI-H1975;  Ten nude male mice of 4 weeks of age (weight 20±1 g) | 24h: IC_50_=104.1 µM;  48h: IC_50_=87.4 µM;  72h: IC_50_=70.6 µM; | ↓ Proliferation;  ↑ Apoptosis;  ↑ FoxO1;  ↓ CCAT1 | ([Hu et al., 2020](#_ENREF_13)) |
| Lung cancer | [Stigmasterol](https://old.tcmsp-e.com/molecule.php?qn=449) | *In vitro* | Lung cancer cells:  PLA-801D, A-549, H661, SK-SEM-1;  Normal lung bronchial epithelial cell line:  BEAS-2B | 5, 10, 20 μg/mL | ↓ Proliferation;  ↑ Apoptosis | ([Dong et al., 2021](#_ENREF_7)) |
| Kidney cancer | Chlorogenic acid | *In vitro* | Human kidney cancer cell:  A498;  Human embryonic kidney cell:  HEK293 | IC_50_ =40±0.98 μM (48 h) | ↓ Proliferation;  ↑ Apoptosis;  ↑ Bax/Bcl-2;  ↓ PI3K/Akt/mTOR pathway | ([Wang et al., 2019](#_ENREF_52)) |
| Breast cancer | Hyperoside | *In vitro; In vivo* | Human breast cancer cell lines:  4T1, MCF-7;  BALB/c mice at 8–10 weeks old (25–30 g) | 0, 25, 50, 100 μM | ↓ NF-κB pathway;  ↓ Migration;  ↑ Apoptosis;  ↓ Bcl-2, XIAP;  ↑ Bax, Caspase-3 | ([Qiu et al., 2019](#_ENREF_40)) |
| Breast cancer | Peel polyphenolic extract (HPP) (hawthorn fruit);  Flesh polyphenolic extract (HFP) (hawthorn fruit) | *In vitro* | Human breast carcinoma cells:  MCF-7, MDA-MB-231;  Normal rat intestinal epithelial cells:  IEC-6 | HPP: IC_50_=88.6 μg/mL; HFP: IC_50_=175.5 μg/mL | ↓ Proliferation;  Cell-cycle arrest at S-phase;  ↑ Apoptosis (mitochondrial pathway);  ↑ Caspase-3, Caspase-9;  ↑ Intracellular ROS | ([Li et al., 2013b](#_ENREF_24)) |
| Colorectal cancer | Hawthorn oligomic procyanidins extracts (HPOE) | *In vitro* | Human colonic cancer cell line:  HCT116 | 250 μg/mL-350 μg/mL | ↓ Proliferation;  Cells cycle arrest at G2/M phase (p53-Cyclin B pathway);  ↑ Apoptosis (mitochondrial and death receptor pathways) | ([Sun et al., 2022](#_ENREF_44)) |
| Lung carcinoma | Extracts (leaves, fruits and seeds of C. aronia) | *In vitro* | Human lung carcinoma cell：A549 | IC_50_=259**±**2.41 µg/mL  (48h)  IC_50_=195**±**2.29 µg/mL (72h) | ↓ Proliferation;  ↑ Apoptosis;  ↓ Bcl-2, PARP-1, Caspase-3  ↑ Bax;  ↓ Migration | ([Omairi et al., 2020](#_ENREF_37)) |
| Colorectal cancer | Ethyl acetate extracts  (*C. azarolus*) | *In vitro* | Human colorectal cell lines:  HT-29, HCT-116 | HT-29 (48 h):  IC_50_=44 mg/mL  HCT-116 (48 h):  IC_50_=32 mg/mL | ↓ Proliferation;  ↑ Apoptosis | ([Mustapha et al., 2016b](#_ENREF_35)) |
| Liver cancer | Hawthorn ethanol extracts | *In vitro* | Human hepatoma carcinoma cell:  HepG2 | 0.2, 0.4, 0.8 g/L | ↓ Proliferation;  ↑ Apoptosis;  ↑ Cleaved-caspase-3, Bax/Bcl-2 | ([Peng et al., 2016](#_ENREF_38)) |
| Endometrial cancer | Isorhamnetin | *In vitro; In vivo* | Human endometrial carcinoma:  Ishikawa cells;  Ishikawa cell-based tumor-bearing mice | IC_50_=37.27 µM | ↑ Apoptosis (mitochondrial and death receptor pathways);  ↑ Endoplasmic reticulum stress-related pathway;  ↓ MMP2, MMP9 | ([Ye et al., 2022](#_ENREF_57)) |
| Hepatocellular carcinoma | Corosolic acid | *In vitro* | Human hepatocellular carcinoma cell lines:  Bel-7402, Bel-7404, and HepG2 | 10, 20 and 30 µM | ↑ ER stress-mediated apoptosis (PERK-eIF2a-ATF4 pathway) | ([Tang et al., 2023](#_ENREF_45)) |
| Liver cancer,  Breast cancer | Triterpenoids-enriched fraction (S9);  Ursolic acid | *In vitro* | Human hepatoma carcinoma cell:  HepG2；  human breast carcinoma cells:  MCF-7, MDA-MB-231 | EC_50_=0.53-1.38 mg/mL | ↓ Proliferation;  Cell cycle arrest at G1 phase;  ↓ PCNA, CDK4, Cyclin D1;  ↑ p21^Waf1/CiP1^;  ↑ Apoptosis (mitochondrial pathway);  ↑ Caspase-9, Caspase-3 | ([Wen et al., 2017](#_ENREF_54)) |
| Colon cancer | Polysaccharide | *In vitro* | Human colon cancer cell:  HCT116 | 125, 250, 500, 1000 µg/mL | ↑ Apoptosis;  Cell cycle arrest at S and G2/M phases;  ↓ Cyclin A1/D1/E1, CDK-1/2 | ([Ma et al., 2020](#_ENREF_29)) |
| Colorectal carcinoma | Orientin | *In vitro* | Human colon cancer cell line:  HT29 | GI_50_=12.55 µM | Cytotoxicity;  ↓ Proliferation;  Cell cycle arrest at G0/G1 phase;  ↓ p53;  ↑ Apoptosis (mitochondrial pathway);  ↑ Intracellular ROS | ([Thangaraj et al., 2019](#_ENREF_48)) |
| Hepatoma | Phenylpropanoid derivatives (fruit of *C.pinnatifida*) | *In vitro* | Human hepatocellular carcinoma cells:  HepG2, Hep3B | 1a: IC_50_=71.46 μM;  1b: IC_50_= 41.37 μM | Cytotoxicity;  ↑ Apoptosis;  Cell cycle arrest at G2/M phase;  ↑ Protective autophagy | ([Guo et al., 2019b](#_ENREF_11)) |
| Breast cancer | Vitexin | *In vitro; In vivo* | Breast cancer cell line of mouse:  EGFR2  Forty female BALB/c mice (weighting 6–8 g) | LD_50_=12 mg/kg | ↑ ATG5, Beclin-1, LC3-II;  ↑ Autophagy | ([Ghazy and Taghi, 2022](#_ENREF_8)) |
| Lung carcinoma | Isoorientin | *in vitro* | Human lung carcinoma cell：A549 | 3-300 μM | ↓ Monocarboxylate transporter (MCT) activity;  ↓ Migration;  ↓ MCTs1/4, CD147, MMP2/9 | ([Huang et al., 2020](#_ENREF_14)) |
| Ovarian cancer | Isorhamnetin | *In vitro; In vivo* | Human ovarian cancer cell lines:  SKOV-3, HO8910;  5-week-old female BALB/c nude mice (18–20 g body weight) | 5, 10, 15, 20 μM;  20 mg/kg | ↓ Proliferation;  ↓ Migration;  ↓ Invasion;  ↓ The growth of OC tumors  ↓ *ESR1* | ([Wang et al., 2022](#_ENREF_51)) |
| Epithelial ovarian cancer | Vitexin | *In vitro; In vivo* | The ovarian cancer cell line:  SKOV-3;  Athymic nude mice (female) | 5, 10, and 20 μM | ↓ Proliferation;  ↓ Invasion;  ↑ Apoptosis;  ↓ VEGFA, VEGFR2;  ↓ p-ERK1/2, p38 | ([Zhao et al., 2020](#_ENREF_62)) |
| Gastric cancer | Vitexin | *In vitro; In vivo* | Human gastric adenocarcinoma cell line:  AGS with *KRAS* mutation (CRL-1739);  Normal human gastric epithelial cell:  GES-1;  Gastric cancer cell line:  SGC-7901 with wild type *KRAS*;  Athymic nude mice (6-week-old females) | 10 μM, 40 μM;  1 mg/kg, 2 mg/kg | ↓ Proliferation;  ↓ Migration;  ↓ Invasion;  ↓ EMT;  ↓ PI3K/AKT/HIF-1α pathway;  ↓ HMGB1 | ([Zhou et al., 2021](#_ENREF_63)) |
| Prostate cancer | Ethanol extract (*C.pinnatifida*);  Chlorogenic acid | *In vitro* | Human prostate cancer cell line:  DU145 | 25, 50 µM | ↓ Proliferation;  ↓ HIF-1α, SPHK-1;  ↓ p-AKT, p-GSK-3β;  ↓ VEGF;  ↓ Angiogenesis  ↓ PCNA, Cyclin D1, CDK4 | ([Lee et al., 2017](#_ENREF_22)) |
| Lung cancer | Isoorientin | *In vitro* | Human lung cancer cell lines:  A549, NCI-H23 and NCI-H460;  Normal lung cell:  IMR-90;  Normal stomach cell:  GES-1 | 46.81 µM | ↑ Apoptosis (mitochondrial pathway);  Cell cycle arrest at G2/M phase;  ↑ ROS levels;  ↓ MAPK/STAT3/NF-κB signaling pathway | ([Xu et al., 2020](#_ENREF_55)) |
| Gastric cancer | Isorhamnetin | *In vitro; In vivo* | Human gastric carcinoma cells:  AGS-1, HGC-27, and HEK293FT;  Male nude recipient mice, 4-6 weeks old | 10, 20, 50, and 100 µM | ↓ Proliferation;  ↓ Migration;  ↑ Apoptosis (mitochondrial pathway);  ↑ ROS | ([Li et al., 2022](#_ENREF_26)) |
| Embryonic cancer | Ursolic acid | *In vitro* | Embryonic carcinoma cells:  NTERA-2, NCCIT | 10 and 20 μM | ↓ Proliferation;  ↑ Cellular and mitochondrial ROS;  Cell cycle arrest at G0/G1 phase;  ↑ Apoptosis | ([Kang et al., 2022](#_ENREF_18)) |
| Ovarian cancer | [Stigmasterol](https://old.tcmsp-e.com/molecule.php?qn=449) | *In vitro* | Clear cell carcinoma cell line:  ES2;  Serous adenocarcinoma cell line:  OV90 | 0, 5, 10, and 20 µg/mL | ↑ Apoptosis;  ↑ ROS;  ↓ Proliferation;  ↓ Migration;  ↓ Angiogenesis;  ↑ Autophagy | ([Bae et al., 2020](#_ENREF_3)) |
| Ovarian cancer | β-Sitosterol | *In vitro* | Human ovarian cancer cells:  ES2, OV90 | 0, 10, 25, and 50 µg/mL | ↓ Proliferation;  ↑ Late cell apoptosis;  ↑ ROS;  ↑ ER stress;  ↓ Migration;  ↓ PI3K/MAPK pathway | ([Bae et al., 2021](#_ENREF_2)) |
| Bladder cancer | Orientin | *In vitro* | Human transitional cell bladder carcinoma cell:  T24 | 0-100 μM | ↓ Proliferation;  Cell cycle arrest;  ↓ Expression of inflammatory mediators;  ↓ NF-κB ;  ↓ Hedgehog signaling pathway;  ↑Apoptosis | ([Tian et al., 2019](#_ENREF_49)) |
| Non-small cell lung cancer | Vitexin | *In vitro; In vivo* | Non-small cell lung cancer cell line:  A549;  Normal human bronchial  epithelial cell line:  16HBE;  Male athymic BALB/c nude mice aged 5-6 weeks | 10, 20, 40 μM | ↑ Apoptosis;  ↓ Proliferation;  ↓ NSCLC tumor growth;  ↓ PI3K/Akt/mTOR signaling pathway;  ↓ Bcl-2/Bax, p-PI3K, p-Akt, p-mTOR;  ↑ Caspase-3 | ([Liu et al., 2019](#_ENREF_27)) |
| Hepatocellular carcinoma | Hyperoside | *In vitro; In vivo* | Human hepatocellular carcinoma cell line:  HepG2;  HCC patients who underwent liver resection surgery | 5, 10, 20, 40, 80 μM | ↓ Proliferation;  Cell cycle arrest;  ↓ PI3K/Akt signaling pathway;  Cell cycle arrest at G1 phase | ([Wei et al., 2021](#_ENREF_53)) |
| Gallbladder cancer | Isorhamnetin | *In vitro; In vivo* | Human gallbladder cancer cell lines:  NOZ, GBC-SD;  Four week old BALB/c nude female mice | NOZ (24h):  IC_50_=162.5 μM;  GBC-SD (24h):  IC_50_=147.1 μM;  NOZ (48h):  IC_50_=103.8 μM;  GBC-SD (48h):  IC_50_=87.27 μM;  NOZ (72h):  IC_50_=81.2 μM;  GBC-SD (72h):  IC_50_=47.52 μM; | ↓ Proliferation;  Cell cycle arrest at G2/M phase;  ↓ Migration;  ↑ Apoptosis;  ↓ PI3K/Akt signaling pathway | ([Zhai et al., 2021](#_ENREF_58)) |
| Breast cancer | Methanolic extract of *C.oxyacantha* berry | *In vitro* | Mammary carcinoma cell lines:  MCF-7, MDA-MB-231;  Peripheral blood mononuclear cells:  PBMCs | MCF-7, MDA-MB-231:  IC_50_=75 μg/mL;  PBMCs:  IC_50_=500-750 μg/mL | Cytotoxicity;  ↓ Proliferation;  Cell cycle arrest at G1/S phase;  ↓ Wnt pathway | ([Kombiyil and Sivasithamparam, 2023](#_ENREF_20)) |
| Gastric cancer | Hawthorn polysaccharide extract | *In vitro* | Human gastric adenocarcinoma cell line:  AGS | 200, 400, 800 μg/mL | ↓ Proliferation;  ↑ Apoptosis;  ↑ Cleaved-caspase-3;  ↑ miR-146a-5p;  ↓ Wnt/β-catenin pathway | ([Li et al., 2021](#_ENREF_25)) |
| Colorectal cancer | Ursolic acid | *In vitro; In vivo* | Human colorectal cancer cell line:  SW620;  Human normal colonic cell line:  NCM460;  Nude mice | 7.5, 15, and 30 μM;  15, 30, and 60 mg/kg | ↓ Proliferation;  ↓ Migration;  ↑ Apoptosis;  Cell cycle arrest at G0/G1 phase;  ↓ Wnt/β-catenin signaling pathway | ([Zhao et al., 2023](#_ENREF_60)) |
| Triple-negative breast cancer | Maslinic acid | *In vitro* | Non-cancerous murine fibroblast cell line:  L292;  Luminal A estrogen positive (ER+) cell line:  MCF7;  TNBC cell lines:  MDA-MB-231, MDA-MB-468 | MDA-MB-231:  IC_50_=38.34 μM;  MDA-MB-468:  IC_50_=49.57 μM;  MCF7:  IC_50_=55.20 μM; | ↑ Apoptosis;  Cell cycle arrest;  ↓ MAPK signaling pathway | ([Jain and Grover, 2020](#_ENREF_17)) |
| Neuroblastoma | Maslinic acid | *In vitro* | Human neuroblastoma cell line:  SHSY-5Y | 0, 10, 40, 80 μM | ↓ Proliferation;  ↑ Caspase-dependent apoptosis;  ↑ ROS;  ↓ Migration;  ↓ Invasion;  ↓ MAPK/ERK signaling pathway | ([Liu et al., 2020](#_ENREF_28)) |
| Oral squamous cell carcinoma | Isorhamnetin | *In vitro* | Oral squamous cell carcinoma cell lines:  HSC-3, HSC-4, PE/CA-PJ15 | HSC-4 (24h):  IC_50_=186.2 μM;  HSC-4 (48h):  IC_50_=115.3 μM;  HSC-4 (72h):  IC_50_=95.88 μM;  HSC-3 (24h):  IC_50_=117.7 μM;  HSC-3 (48h):  IC_50_=62.99 μM;  HSC-3 (72h):  IC_50_=40.36 μM; | ↓ Proliferation;  Cell cycle arrest at G2/M phase;  ↓ Cyclin B1, CDC2;  ↓ Migration;  ↑ Apoptosis;  ↑ Phosphorylated ERK cascades;  ↑ Intracellular ROS levels | ([Chen et al., 2021](#_ENREF_5)) |
| Hepatoma carcinoma | Orientin | *In vitro* | Hepatocellular carcinoma cell lines:  HepG2, Huh7 | 50, 100 μM | ↓ Proliferation;  ↓ Migration;  ↓ NF-κB signaling pathway | ([Tao et al., 2023](#_ENREF_47)) |
| Lung cancer | Chlorogenic acid | *In vitro; In vivo* | Human lung adenocarcinoma cell line:  A549;  Six weeks male BALB/c nude mice | 0-800 μM | ↓ Proliferation;  Cell cycle arrest;  ↓ cIAP1, cIAP2 of the NF-κB signaling pathway;  ↓ Migration | ([Wang et al., 2020](#_ENREF_50)) |
| Hepatocellular carcinoma | Vitexin | *In vitro* | Human HCC cell lines:  HepG2, Hep3B, HCCLM3, and PLC/PRF5 | 0-50 μM | ↓ STAT3 signaling cascade;  ↓ Proliferation;  ↓ Invasion | ([Lee et al., 2020](#_ENREF_21)) |
| Human oral cancer | Vitexin | *In vitro* | Human oral cancer cell line:  OC2 | 0, 12.5, 25, 50, 100 mM | ↓ Proliferation;  ↑ Plasminogen activator inhibitor 1 (PAI-1) accumulation;  ↓ MMP-2;  ↑ p53, p21^WAF1^, Bax;  ↓ Metastasis;  ↑ Apoptosis (p53-dependent pathway) | ([Yang et al., 2013](#_ENREF_56)) |

Ahn, K.S., Hahm, M.S., Park, E.J., Lee, H.K., and Kim, I.H. (1998). Corosolic acid isolated from the fruit of Crataegus pinnatifida var. psilosa is a protein kinase C inhibitor as well as a cytotoxic agent. *Planta Med* 64(5)**,** 468-470. doi: 10.1055/s-2006-957487.

Bae, H., Park, S., Ham, J., Song, J., Hong, T., Choi, J.H., et al. (2021). ER-Mitochondria Calcium Flux by β-Sitosterol Promotes Cell Death in Ovarian Cancer. *Antioxidants (Basel)* 10(10). doi: 10.3390/antiox10101583.

Bae, H., Song, G., and Lim, W. (2020). Stigmasterol Causes Ovarian Cancer Cell Apoptosis by Inducing Endoplasmic Reticulum and Mitochondrial Dysfunction. *Pharmaceutics* 12(6). doi: 10.3390/pharmaceutics12060488.

Belščak-Cvitanović, A., Durgo, K., Bušić, A., Franekić, J., and Komes, D. (2014). Phytochemical attributes of four conventionally extracted medicinal plants and cytotoxic evaluation of their extracts on human laryngeal carcinoma (HEp2) cells. *J Med Food* 17(2)**,** 206-217. doi: 10.1089/jmf.2013.0071.

Chen, Q., Song, S., Wang, Z., Shen, Y., Xie, L., Li, J., et al. (2021). Isorhamnetin induces the paraptotic cell death through ROS and the ERK/MAPK pathway in OSCC cells. *Oral Dis* 27(2)**,** 240-250. doi: 10.1111/odi.13548.

Diao, T.T., Zhang, Y.C., Lv, W., and Min, Q. (2019). Experimental study on inhibitory effect of hawthorn leaves flavonoids on human glioblastoma U87 cells. *Chinese Pharmacological Bulletin* 35(10)**,** 1448-1452.

Dong, Y., Chen, C., Chen, C., Zhang, C., Zhang, L., Zhang, Y., et al. (2021). Stigmasterol inhibits the progression of lung cancer by regulating retinoic acid-related orphan receptor C. *Histol Histopathol* 36(12)**,** 1285-1299. doi: 10.14670/hh-18-388.

Ghazy, E., and Taghi, H.S. (2022). The Autophagy-Inducing Mechanisms of Vitexin, Cinobufacini, and Physalis alkekengi Hydroalcoholic Extract against Breast Cancer in vitro and in vivo. *J Gastrointest Cancer* 53(3)**,** 592-596. doi: 10.1007/s12029-021-00668-0.

Guo, R., Lin, B., Shang, X.Y., Zhou, L., Yao, G.D., Huang, X.X., et al. (2018). Phenylpropanoids from the fruit of Crataegus pinnatifida exhibit cytotoxicity on hepatic carcinoma cells through apoptosis induction. *Fitoterapia* 127**,** 301-307. doi: 10.1016/j.fitote.2018.03.003.

Guo, R., Lv, T.M., Shang, X.Y., Yao, G.D., Lin, B., Wang, X.B., et al. (2019a). Racemic neolignans from Crataegus pinnatifida: Chiral resolution, configurational assignment, and cytotoxic activities against human hepatoma cells. *Fitoterapia* 137**,** 104287. doi: 10.1016/j.fitote.2019.104287.

Guo, R., Shang, X.Y., Lv, T.M., Yao, G.D., Lin, B., Wang, X.B., et al. (2019b). Phenylpropanoid derivatives from the fruit of Crataegus pinnatifida Bunge and their distinctive effects on human hepatoma cells. *Phytochemistry* 164**,** 252-261. doi: 10.1016/j.phytochem.2019.05.005.

Guo, W., Yu, H., Zhang, L., Chen, X., Liu, Y., Wang, Y., et al. (2019c). Effect of hyperoside on cervical cancer cells and transcriptome analysis of differentially expressed genes. *Cancer Cell Int* 19**,** 235. doi: 10.1186/s12935-019-0953-4.

Hu, Z., Zhao, P., and Xu, H. (2020). Hyperoside exhibits anticancer activity in non‑small cell lung cancer cells with T790M mutations by upregulating FoxO1 via CCAT1. *Oncol Rep* 43(2)**,** 617-624. doi: 10.3892/or.2019.7440.

Huang, H.K., Lee, S.Y., Huang, S.F., Lin, Y.S., Chao, S.C., Huang, S.F., et al. (2020). Isoorientin Decreases Cell Migration via Decreasing Functional Activity and Molecular Expression of Proton-Linked Monocarboxylate Transporters in Human Lung Cancer Cells. *Am J Chin Med* 48(1)**,** 201-222. doi: 10.1142/s0192415x20500111.

Huang, X.X., Zhou, C.C., Li, L.Z., Li, F.F., Lou, L.L., Li, D.M., et al. (2013a). The cytotoxicity of 8-O-4' neolignans from the seeds of Crataegus pinnatifida. *Bioorg Med Chem Lett* 23(20)**,** 5599-5604. doi: 10.1016/j.bmcl.2013.08.045.

Huang, X.X., Zhou, C.C., Li, L.Z., Peng, Y., Lou, L.L., Liu, S., et al. (2013b). Cytotoxic and antioxidant dihydrobenzofuran neolignans from the seeds of Crataegus pinnatifida. *Fitoterapia* 91**,** 217-223. doi: 10.1016/j.fitote.2013.09.011.

Jain, R., and Grover, A. (2020). Maslinic acid differentially exploits the MAPK pathway in estrogen-positive and triple-negative breast cancer to induce mitochondrion-mediated, caspase-independent apoptosis. *Apoptosis* 25(11-12)**,** 817-834. doi: 10.1007/s10495-020-01636-y.

Kang, D.Y., Sp, N., Jang, K.J., Jo, E.S., Bae, S.W., and Yang, Y.M. (2022). Antitumor Effects of Natural Bioactive Ursolic Acid in Embryonic Cancer Stem Cells. *J Oncol* 2022**,** 6737248. doi: 10.1155/2022/6737248.

Kao, E.S., Wang, C.J., Lin, W.L., Chu, C.Y., and Tseng, T.H. (2007). Effects of polyphenols derived from fruit of Crataegus pinnatifida on cell transformation, dermal edema and skin tumor formation by phorbol ester application. *Food Chem Toxicol* 45(10)**,** 1795-1804. doi: 10.1016/j.fct.2007.03.016.

Kombiyil, S., and Sivasithamparam, N.D. (2023). In Vitro Anti-cancer Effect of Crataegus oxyacantha Berry Extract on Hormone Receptor Positive and Triple Negative Breast Cancers via Regulation of Canonical Wnt Signaling Pathway. *Appl Biochem Biotechnol* 195(4)**,** 2687-2708. doi: 10.1007/s12010-021-03724-4.

Lee, J.H., Mohan, C.D., Shanmugam, M.K., Rangappa, S., Sethi, G., Siveen, K.S., et al. (2020). Vitexin abrogates invasion and survival of hepatocellular carcinoma cells through targeting STAT3 signaling pathway. *Biochimie* 175**,** 58-68. doi: 10.1016/j.biochi.2020.05.006.

Lee, M.S., Lee, S.O., Kim, K.R., and Lee, H.J. (2017). Sphingosine Kinase-1 Involves the Inhibitory Action of HIF-1α by Chlorogenic Acid in Hypoxic DU145 Cells. *Int J Mol Sci* 18(2). doi: 10.3390/ijms18020325.

Li, L.Z., Peng, Y., Niu, C., Gao, P.Y., Huang, X.X., Mao, X.L., et al. (2013a). Isolation of cytotoxic compounds from the seeds of Crataegus pinnatifida. *Chin J Nat Med* 11(4)**,** 411-414. doi: 10.1016/s1875-5364(13)60061-8.

Li, T., Zhu, J., Guo, L., Shi, X., Liu, Y., and Yang, X. (2013b). Differential effects of polyphenols-enriched extracts from hawthorn fruit peels and fleshes on cell cycle and apoptosis in human MCF-7 breast carcinoma cells. *Food Chem* 141(2)**,** 1008-1018. doi: 10.1016/j.foodchem.2013.04.050.

Li, X.P., Li, W., Gao, F.F., Huang, C.G., He, T.Y., and Sun, Z.J. (2021). Up-regulation of miR-146a-5p by hawthorn polysaccharide extract inhibits Wnt/β-catenin signaling pathway to affect proliferation and apoptosis of gastric cancer cells. *Anhui Medical and Pharmaceutical Journal* 25(02)**,** 326-330.

Li, Y., Fan, B., Pu, N., Ran, X., Lian, T., Cai, Y., et al. (2022). Isorhamnetin Suppresses Human Gastric Cancer Cell Proliferation through Mitochondria-Dependent Apoptosis. *Molecules* 27(16). doi: 10.3390/molecules27165191.

Liu, X., Jiang, Q., Liu, H., and Luo, S. (2019). Vitexin induces apoptosis through mitochondrial pathway and PI3K/Akt/mTOR signaling in human non-small cell lung cancer A549 cells. *Biol Res* 52(1)**,** 7. doi: 10.1186/s40659-019-0214-y.

Liu, Y., Lu, H., Dong, Q., Hao, X., and Qiao, L. (2020). Maslinic acid induces anticancer effects in human neuroblastoma cells mediated via apoptosis induction and caspase activation, inhibition of cell migration and invasion and targeting MAPK/ERK signaling pathway. *AMB Express* 10(1)**,** 104. doi: 10.1186/s13568-020-01035-1.

Ma, L., Xu, G.Y.B., Tang, X.Y., Zhang, C., Zhao, W., Wang, J., et al. (2020). Anti-cancer potential of polysaccharide extracted from hawthorn (Crataegus.) on human colon cancer cell line HCT116 via cell cycle arrest and apoptosis. *Journal of Functional Foods* 64. doi: 10.1016/j.jff.2019.103677.

Maldonado-Cubas, J., Albores-Méndez, E.M., San Martín-Martínez, E., Quiroz-Reyes, C.N., González-Córdova, G.E., and Casañas-Pimentel, R.G. (2020). Mexican hawthorn (Crataegus gracilior J. B. Phipps) stems and leaves induce cell death on breast cancer cells. *Nutr Cancer* 72(8)**,** 1411-1421. doi: 10.1080/01635581.2019.1678657.

Min, B.S., Kim, Y.H., Lee, S.M., Jung, H.J., Lee, J.S., Na, M.K., et al. (2000). Cytotoxic triterpenes from Crataegus pinnatifida. *Arch Pharm Res* 23(2)**,** 155-158. doi: 10.1007/bf02975505.

Mohammedsaeed, A.A., and Mohamad, T.S. (2023). Inhibitory and anti-Cancer Effects of Crataegus azarolus Extracts on Gastric Cancer Cell Line (AGS). *ZANCO Journal of Pure and Applied Sciences* 35(2)**,** 211-220. doi: 10.21271/zjpas.35.2.22.

Mustapha, N., Bzéouich, I.M., Ghedira, K., Hennebelle, T., and Chekir-Ghedira, L. (2015). Compounds isolated from the aerial part of Crataegus azarolus inhibit growth of B16F10 melanoma cells and exert a potent inhibition of the melanin synthesis. *Biomed Pharmacother* 69**,** 139-144. doi: 10.1016/j.biopha.2014.11.010.

Mustapha, N., Mokdad-Bzéouich, I., Maatouk, M., Ghedira, K., Hennebelle, T., and Chekir-Ghedira, L. (2016a). Antitumoral, antioxidant, and antimelanogenesis potencies of Hawthorn, a potential natural agent in the treatment of melanoma. *Melanoma Res* 26(3)**,** 211-222. doi: 10.1097/cmr.0000000000000240.

Mustapha, N., Pinon, A., Limami, Y., Simon, A., Ghedira, K., Hennebelle, T., et al. (2016b). Crataegus azarolus Leaves Induce Antiproliferative Activity, Cell Cycle Arrest, and Apoptosis in Human HT-29 and HCT-116 Colorectal Cancer Cells. *J Cell Biochem* 117(5)**,** 1262-1272. doi: 10.1002/jcb.25416.

Najafipour, R., Momeni, A.M., Mirmazloomi, Y., and Moghbelinejad, S. (2022). Vitexin Induces Apoptosis in MCF-7 Breast Cancer Cells through the Regulation of Specific miRNAs Expression. *Int J Mol Cell Med* 11(3)**,** 197-206. doi: 10.22088/ijmcm.Bums.11.3.197.

Omairi, I., Kobeissy, F., and Nasreddine, S. (2020). Anti-Oxidant, Anti-Hemolytic Effects of Crataegus aronia Leaves and Its Anti- Proliferative Effect Enhance Cisplatin Cytotoxicity in A549 Human Lung Cancer Cell Line. *Asian Pac J Cancer Prev* 21(10)**,** 2993-3003. doi: 10.31557/apjcp.2020.21.10.2993.

Peng, F.H., Ma, X., and Hu, X.Y. (2016). Effect of Hawthorn Extract on Apoptosis and Related Factors of HepG2 Cells. *Chinese Journal of Experimental Traditional Medical Formulae* 22(07)**,** 169-172. doi: 10.13422/j.cnki.syfjx.2016070169.

Qiao, A., Wang, Y., Xiang, L., Zhang, Z., and He, X. (2015). Novel triterpenoids isolated from hawthorn berries functioned as antioxidant and antiproliferative activities. *Journal of Functional Foods* 13**,** 308-313.

Qiu, J., Zhang, T., Zhu, X., Yang, C., Wang, Y., Zhou, N., et al. (2019). Hyperoside Induces Breast Cancer Cells Apoptosis via ROS-Mediated NF-κB Signaling Pathway. *Int J Mol Sci* 21(1). doi: 10.3390/ijms21010131.

Rodrigues, S., Calhelha, R.C., Barreira, J.C.M., Dueñas, M., Carvalho, A.M., Abreu, R.M.V., et al. (2012). Crataegus monogyna buds and fruits phenolic extracts: Growth inhibitory activity on human tumor cell lines and chemical characterization by HPLC–DAD–ESI/MS. *Food Research International* 49(1)**,** 516-523. doi: 10.1016/j.foodres.2012.07.046.

Shang, X.Y., Guo, R., Yu, X.Q., Lin, B., Huang, X.X., Yao, G.D., et al. (2020). Enantiomeric 8-O-4'-type neolignans from Crataegus pinnatifida exhibit cytotoxic effect via apoptosis and autophagy in Hep3B cells. *Bioorg Chem* 104**,** 104267. doi: 10.1016/j.bioorg.2020.104267.

Sun, J., Gao, G., Gao, Y., Xiong, L., Li, X., Guo, J., et al. (2013). Experimental research on the in vitro antitumor effects of Crataegus sanguinea. *Cell Biochem Biophys* 67(1)**,** 207-213. doi: 10.1007/s12013-013-9535-6.

Sun, Y.S., Wang, Z.W., Gao, Z., Zhao, W., Thakur, K., Zhong, Q., et al. (2022). Proanthocyanidin oligomers extract from hawthorn mediates cell cycle arrest, apoptosis, and lysosome vacuolation on HCT116 cells. *Curr Res Food Sci* 5**,** 904-917. doi: 10.1016/j.crfs.2022.05.009.

Tang, F., Peng, Y., Liu, J., Gao, W., and Xu, Y. (2023). Integrating network pharmacology and experimental models to examine the mechanisms of corosolic acid in preventing hepatocellular carcinoma progression through activation PERK-eIF2a-ATF4 signaling. *Naunyn Schmiedebergs Arch Pharmacol* 396(12)**,** 3671-3682. doi: 10.1007/s00210-023-02560-z.

Tang, S.Y., C., H.G., Yang, F.G., Xu, D.W., and Li, C.H. (2010). Effect of Chinese hawthorn leaf flavone on human monocaryon leukemia cell proliferation. 21(03)**,** 269-270. doi: 10.19378/j.issn.1003-9783.2010.03.018.

Tao, J.Y., Li, J., Wan, L., Dong, B.Z., Yu, Y.J., Liu, Y.M., et al. (2023). Orientin regulates the proliferation and migration of hepatocellular carcinoma cells. *Naunyn Schmiedebergs Arch Pharmacol* 396(10)**,** 2519-2528. doi: 10.1007/s00210-023-02472-y.

Thangaraj, K., Balasubramanian, B., Park, S., Natesan, K., Liu, W., and Manju, V. (2019). Orientin Induces G0/G1 Cell Cycle Arrest and Mitochondria Mediated Intrinsic Apoptosis in Human Colorectal Carcinoma HT29 Cells. *Biomolecules* 9(9). doi: 10.3390/biom9090418.

Tian, F., Tong, M., Li, Z., Huang, W., Jin, Y., Cao, Q., et al. (2019). The Effects of Orientin on Proliferation and Apoptosis of T24 Human Bladder Carcinoma Cells Occurs Through the Inhibition of Nuclear Factor-kappaB and the Hedgehog Signaling Pathway. *Med Sci Monit* 25**,** 9547-9554. doi: 10.12659/msm.919203.

Wang, L., Du, H., and Chen, P. (2020). Chlorogenic acid inhibits the proliferation of human lung cancer A549 cell lines by targeting annexin A2 in vitro and in vivo. *Biomed Pharmacother* 131**,** 110673. doi: 10.1016/j.biopha.2020.110673.

Wang, M., Xu, Z., Cai, Q., Deng, Y., Shi, W., Zhou, H., et al. (2022). Isorhamnetin inhibits progression of ovarian cancer by targeting ESR1. *Ann Transl Med* 10(22)**,** 1216. doi: 10.21037/atm-22-5064.

Wang, X., Liu, J., Xie, Z., Rao, J., Xu, G., Huang, K., et al. (2019). Chlorogenic acid inhibits proliferation and induces apoptosis in A498 human kidney cancer cells via inactivating PI3K/Akt/mTOR signalling pathway. *J Pharm Pharmacol* 71(7)**,** 1100-1109. doi: 10.1111/jphp.13095.

Wei, S., Sun, Y., Wang, L., Zhang, T., Hu, W., Bao, W., et al. (2021). Hyperoside suppresses BMP-7-dependent PI3K/AKT pathway in human hepatocellular carcinoma cells. *Ann Transl Med* 9(15)**,** 1233. doi: 10.21037/atm-21-2980.

Wen, L., Guo, R., You, L., Abbasi, A.M., Li, T., Fu, X., et al. (2017). Major triterpenoids in Chinese hawthorn "Crataegus pinnatifida" and their effects on cell proliferation and apoptosis induction in MDA-MB-231 cancer cells. *Food Chem Toxicol* 100**,** 149-160. doi: 10.1016/j.fct.2016.12.032.

Xu, W.T., Shen, G.N., Li, T.Z., Zhang, Y., Zhang, T., Xue, H., et al. (2020). Isoorientin induces the apoptosis and cell cycle arrest of A549 human lung cancer cells via the ROS‑regulated MAPK, STAT3 and NF‑κB signaling pathways. *Int J Oncol* 57(2)**,** 550-561. doi: 10.3892/ijo.2020.5079.

Yang, S.H., Liao, P.H., Pan, Y.F., Chen, S.L., Chou, S.S., and Chou, M.Y. (2013). The novel p53-dependent metastatic and apoptotic pathway induced by vitexin in human oral cancer OC2 cells. *Phytother Res* 27(8)**,** 1154-1161. doi: 10.1002/ptr.4841.

Ye, L., Ma, R.H., Zhang, X.X., Thakur, K., Zhang, J.G., Khan, M.R., et al. (2022). Isorhamnetin Induces Apoptosis and Suppresses Metastasis of Human Endometrial Carcinoma Ishikawa Cells via Endoplasmic Reticulum Stress Promotion and Matrix Metalloproteinase-2/9 Inhibition In Vitro and In Vivo. *Foods* 11(21). doi: 10.3390/foods11213415.

Zhai, T., Zhang, X., Hei, Z., Jin, L., Han, C., Ko, A.T., et al. (2021). Isorhamnetin Inhibits Human Gallbladder Cancer Cell Proliferation and Metastasis via PI3K/AKT Signaling Pathway Inactivation. *Front Pharmacol* 12**,** 628621. doi: 10.3389/fphar.2021.628621.

Zhang, Y., Li, H.W., Sun, J.P., Zhang, Y.C., He, S.Z., and Yang, B.F. (2004). Extraction and isolation of total flavonoids from hawthorn fruit and their antitumor activity in vitro. *Chinese Traditional and Herbal Drugs* 07**,** 72-74.

Zhao, H., Tang, S., Tao, Q., Ming, T., Lei, J., Liang, Y., et al. (2023). Ursolic Acid Suppresses Colorectal Cancer by Down-Regulation of Wnt/β-Catenin Signaling Pathway Activity. *J Agric Food Chem* 71(9)**,** 3981-3993. doi: 10.1021/acs.jafc.2c06775.

Zhao, P., Guo, R., Zhang, Y.Y., Zhang, H., Yao, G.D., Lin, B., et al. (2019). Phenylpropanoid and dibenzofuran derivatives from Crataegus pinnatifida with antiproliferative activities on hepatoma cells. *Bioorg Chem* 93**,** 103354. doi: 10.1016/j.bioorg.2019.103354.

Zhao, S., Guan, X., Hou, R., Zhang, X., Guo, F., Zhang, Z., et al. (2020). Vitexin attenuates epithelial ovarian cancer cell viability and motility in vitro and carcinogenesis in vivo via p38 and ERK1/2 pathways related VEGFA. *Ann Transl Med* 8(18)**,** 1139. doi: 10.21037/atm-20-5586.

Zhou, P., Zheng, Z.H., Wan, T., Wu, J., Liao, C.W., and Sun, X.J. (2021). Vitexin Inhibits Gastric Cancer Growth and Metastasis through HMGB1-mediated Inactivation of the PI3K/AKT/mTOR/HIF-1α Signaling Pathway. *J Gastric Cancer* 21(4)**,** 439-456. doi: 10.5230/jgc.2021.21.e40.

Zhu, R.M. (2012). Effect of water extracts from Hawthorn on Ca-2 cells proliferation. *China Medicine and Pharmacy* 2(23)**,** 39-40.

Żurek, N., Karatsai, O., Rędowicz, M.J., and Kapusta, I.T. (2021). Polyphenolic Compounds of Crataegus Berry, Leaf, and Flower Extracts Affect Viability and Invasive Potential of Human Glioblastoma Cells. *Molecules* 26(9). doi: 10.3390/molecules26092656.
